# Transmembrane transport process and endoplasmic reticulum function facilitate the role of gene *cel1b* in cellulase production of *Trichoderma reesei*

**DOI:** 10.1186/s12934-022-01809-1

**Published:** 2022-05-19

**Authors:** Ai-Ping Pang, Yongsheng Luo, Xin Hu, Funing Zhang, Haiyan Wang, Yichen Gao, Samran Durrani, Chengcheng Li, Xiaotong Shi, Fu-Gen Wu, Bing-Zhi Li, Zuhong Lu, Fengming Lin

**Affiliations:** 1 State Key Laboratory of Bioelectronics, School of Biological Science and Medical Engineering, Southeast University, Nanjing, China; 2 International Innovation Center for Forest Chemicals and Materials and Jiangsu Co-Innovation Center for Efficient Processing and Utilization of Forest Resources, Nanjing Forestry University, Nanjing, 210037 China; 3 Key Laboratory of Systems Bioengineering (Ministry of Education), School of Chemical Engineering and Technology, Tianjin University, Tianjin, China

**Keywords:** Filamentous fungus, β-glucosidase, *cel1b*, Sugar transporters, Protein export, Protein processing, Endoplasmic reticulum

## Abstract

**Background:**

A total of 11 β-glucosidases are predicted in the genome of *Trichoderma reesei*, which are of great importance for regulating cellulase biosynthesis. Nevertheless, the relevant function and regulation mechanism of each β-glucosidase remained unknown.

**Results:**

We evidenced that overexpression of *cel1b* dramatically decreased cellulase synthesis in *T. reesei* RUT-C30 both at the protein level and the mRNA level. In contrast, the deletion of *cel1b* did not noticeably affect cellulase production. Protein CEL1B was identified to be intracellular, being located in vacuole and cell membrane. The overexpression of *cel1b* reduced the intracellular pNPGase activity and intracellular/extracellular glucose concentration without inducing carbon catabolite repression. On the other hand, RNA-sequencing analysis showed the transmembrane transport process and endoplasmic reticulum function were affected noticeably by overexpressing *cel1b*. In particular, some important sugar transporters were notably downregulated, leading to a compromised cellular uptake of sugars including glucose and cellobiose.

**Conclusions:**

Our data suggests that the cellulase inhibition by *cel1b* overexpression was not due to the β-glucosidase activity, but probably the dysfunction of the cellular transport process (particularly sugar transport) and endoplasmic reticulum (ER). These findings advance the knowledge of regulation mechanism of cellulase synthesis in filamentous fungi, which is the basis for rationally engineering *T. reesei* strains to improve cellulase production in industry.

**Supplementary Information:**

The online version contains supplementary material available at 10.1186/s12934-022-01809-1.

## Background

Cellulose widely exists in nature and waste materials such as leaves, grass, wood, municipal wastes, agricultural wastes, etc. Bioconversion of cellulose by cellulase to fermentable sugars for biomass-based biorefinery is a sustainable and environment-friendly measure to make the best of cellulose [1–6]. The complete decomposition of cellulose needs sequential and synergistic actions of fungal cellulase, mainly including endoglucanase (EG; EC 3.2.1.4) attacking cellulose in an endo-acting manner with a strong affinity towards the soluble derivatives of cellulose, cellobiohydrolase (CBH; EC 3.2.1.91) acting like exoenzymes to release cellobiose from crystalline cellulose as the main product, and β-glucosidase (BGL; EC 3.2.1.21) cleaving cellobiose to glucose [7–11]. Among these three major components of cellulase, β-glucosidases take a crucial part in the regulation of cellulase biosynthesis in filamentous fungi, being an object of major research efforts [12–18].

*Trichoderma reesei*, the work horse for cellulase production in industry, contains 11 genes encoding β-glucosidases which are classified into glycoside hydrolase (GH) families 1 (*cel1a* and *cel1b*) and 3 (*cel3a*, *cel3b*, *cel3c*, *cel3d*, *cel3e*, *cel3f*, *cel3g*, *cel3h*, and *cel3j*) on the basis of their sequence identity and structural similarity [19, 20]. They are distributed at different genomic sites with genes *cel3e*, *cel3f* and *cel3g* in chromosome I, *cel1b* in chromosome II, *cel3a*, *cel1a* and *cel3d* in chromosome III, *cel3h*, *cel3j* and *cel3c* in chromosome IV, and *cel3b* in chromosome VI [21]. Their effects on cellulase production, and their cellular distribution and secretion have been comprehensively studied, identifying that β-glucosidases CEL1A, CEL3B, CEL3E, CEL3F, CEL3G, CEL3H, and CEL3J are extracellular, CEL3C and CEL3D intracellular, CEL1B unknown [22]. The well characterized β-glucosidase was CEL3A, responsible for most of the extracellular β-glucosidase activity [23]. β-glucosidase *cel3a* is essential for rapid cellulase induction in *T. reesei* [24], whose overexpression can enhance cellulase production [25–28]. It is reported that deletion of *cel3g* significantly improved cellulase production on lactose due to increased transcription of lactose permease that might be involved in lactose transport [29]. In spite of these efforts on researching *T. reesei* β-glucosidases, the varied role of each β-glucosidase plays in cellulase biosynthesis remains to be established.

β-glucosidase CEL1B was predicted to be an intracellular β-glucosidase, showing the lowest substrate specific activity against pNPG, G3, G4 and Gen compared to other β-glucosidases [30]. It was highly induced in *T. reesei* cultivated on cellulose or sophorose together with *cel1a* [31]. The absence of gene *cel1b* or *cel1a* delayed the cellulase expression induced by cellulose, indicating they participate in the rapid cellulase induction [32]. CEL1B and CEL1A are required for cellulase induction by lactose, which are functionally equivalent to each other [33]. The mRNA level of *cel1b* was increased in *T. reesei* Δ*cre1* with high cellulase production, and was downregulated in both *T. reesei* Δ*xyr1* [34] and ZC121 [35] with pretty low cellulase production on cellulose. More interestingly, gene *cel1b* is predicted to contain multiple binding sites for several transcriptional factors relevant to cellulase biosynthesis, including 6 for CRE1 [34], 5 for Xyr1 [36], 6 for Ace1 [37], 3 for Ace3 [38], and 5 for Hap2/3/5 complex [39]. Altogether, *cel1b* is significantly involved in the regulatory network of cellulase synthesis. Therefore, it is highly desirable to investigate the detailed function and regulation mechanism of *cel1b* in cellulase production in *T. reesei*.

In this study, we confirmed that the overexpression of predicted β-glucosidase *cel1b* in *T. reesei* RUT-C30 dramatically decreased cellulase production on cellulose using three different promoters. We explored extensively the underlying mechanism of this phenomenon. We investigated the effect of *cel1b* deletion on cellulase production, and its cellular distribution. We exclude the possibility that the cellulase repression of *cel1b* overexpression was because of its β-glucosidase activity. In contrast, transcriptome analysis showed that the overexpression of *cel1b* significantly impacted the cellular transport process and ER function, which was further confirmed by several lines of evidences.

## Results

### Overexpression of *cel1b* dramatically decreased cellulase production of *T. reesei* on cellulose

**Fig. 1:**
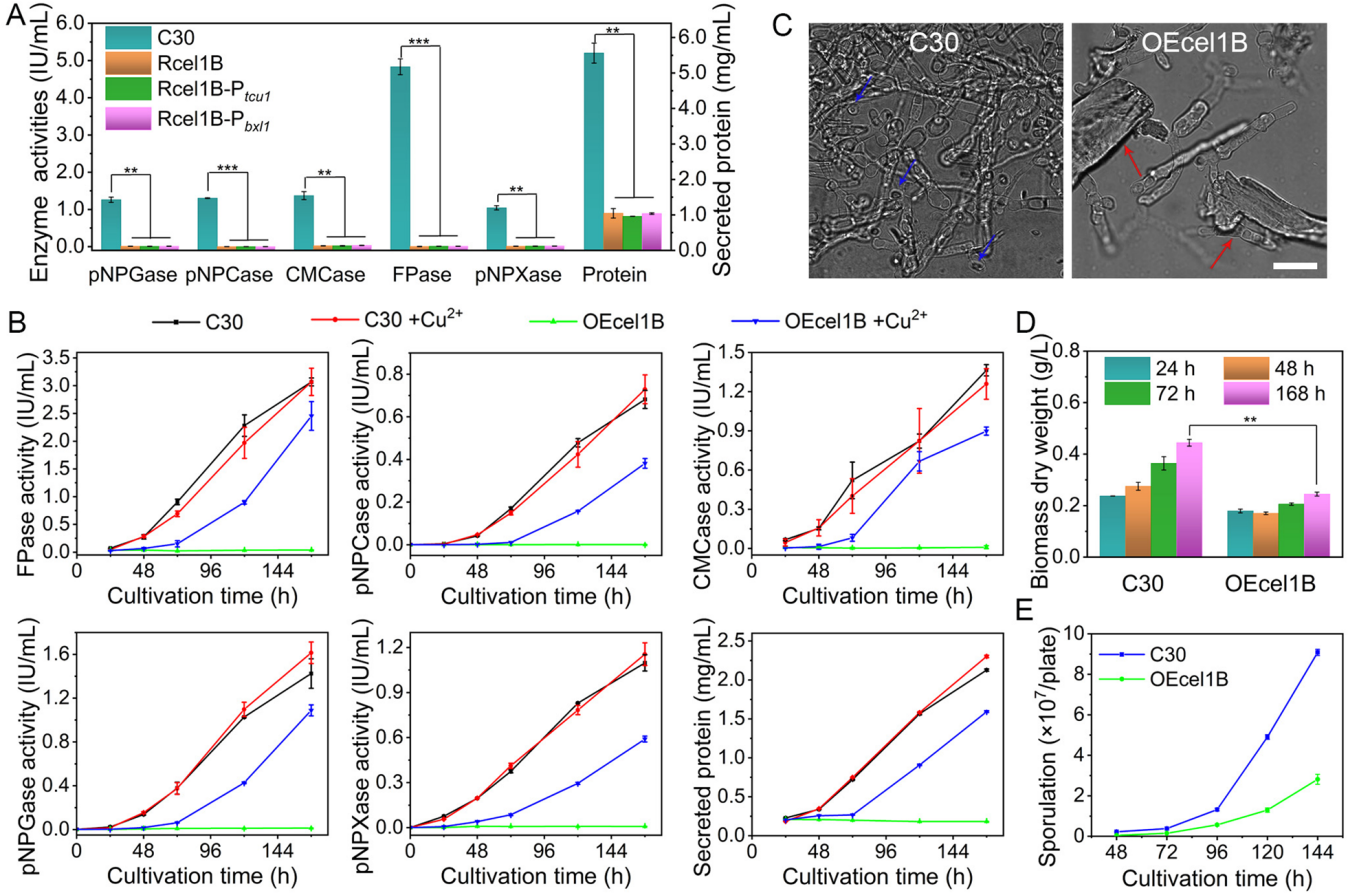
The effects of *cel1b* overexpression on cellulase activities, morphology, cell growth, and sporulation. **A** Cellulase activities and protein secretion at 168 h for *T. reesei* RUT-C30 and *cel1b*-overexpressing strains Rcel1B, OEcel1B-P_*tcu1*_ (OEcel1B), and OEcel1B-P_*bxl1*_ using promoter *cbh1*, *tcu1* and *bxl1*, respectively. **B** Cellulase activities and protein secretion for *T. reesei* RUT-C30 and OEcel1B with or without 20 µM Cu^2+^. **C** Cell morphology at 168. Blue and red arrows indicated the spores and insoluble cellulose, respectively. Scale bar = 20 μm. **D** Cell growth. **E** Sporulation. All strains were cultured in TMM + 2% cellulose. Data are represented as the mean of three independent experiments, and error bars express the standard deviations. Asterisks indicate significant differences (*p < 0.05, **p < 0.01, and ***p < 0.001) as assessed by Student’s *t* test

The mRNA levels of *cel1b* were 3.30, 1.11, and 3.17 at 24 h, 72 h, and 120 h respectively in *T. reesei* RUT-C30 grown on cellulose for cellulase production (Additional file 1: Fig. S1). This data was consistent with the reported finding that *cel1b* was highly induced in *T. reesei* cultivated on cellulose or sophorose together with *cel1a* [31], whose transcriptional levels were only second to the major extracellular β-glucosidase CEL3A [22], demonstrating that *cel1b* might be actively involved in the cellulase biosynthesis in *T. reesei*. Our previous study has shown that *cel1b* overexpression using an inducible *cbh1* promoter led to a dramatic decrease in cellulase production on cellulose as observed in *T. reesei* Rcel1B, while the overexpression of other β-glucosidases did not [22]. We wondered if the decrease on cellulase production was due to that the promoter *cbh1* was too strong, which was considered as the strongest promoter in *T. reesei* on cellulose [40, 41]. Therefore, another two weaker promoters *tcu1* [42] and *bxl1* [43] were harnessed for *cel1b* overexpression (Additional file 1: Fig. S2A) in *T. reesei* RUT-C30, to obtain mutant strains OEcel1B-P_*tcu1*_ and OEcel1B-P_*bxl1*_ respectively. Promoter *tcu1*, which was highly responsive to copper, was constitutively expressed in the absence of copper, and was repressed in the presence of copper. Promoter *bxl1* was an inducible promoter by xylobiose [44, 45]. The effect of *cel1b* overexpression under all three different promoters on cellulase production at 168 h using cellulose as the carbon source was investigated by comparison with the parent strain *T. reesei* RUT-C30 (Fig. 1A). Regardless of different promoters, the cellulase activities pNPGase, pNPCase, CMCase and the total filter paper FPase were all decreased dramatically by at least 97% in all three *cel1b*-overexpressing strains Rcel1B, OEcel1B-P_*tcu1*_ and OEcel1B-P_*bxl1*_. The hemicellulase activity pNPXase was also declined significantly in strains Rcel1B, OEcel1B-P_*tcu1*_ and OEcel1B-P_*bxl1*_, which was only 1.07%, 1.27% and 1.61% of that in RUT-C30 respectively. Correspondingly, the extracellular protein concentration in the supernatants of all three mutant strains was lower than that of RUT-C30. This phenomenon was not colony specific, as the enzyme activities and protein concentration of the other three random selected mutant transformants for each promoter were significantly decreased (Additional file 1: Fig. S3). All these demonstrated that overexpression of *cel1b* in *T. reesei* inhibited the cellulase production severely, which was not dependent on the strength of promoters and the overexpression degree of *cel1b*. The transformant OEcel1B-P_*tcu1*_ was selected for further study, which was named OEcel1B in the following study if not specifically indicated.

To exclude the possibility that the decreased cellulase production was resulted from random integration of *cel1b* into *T. reesei* genome, we repressed the overexpression of the inserted *cel1b* in *T. reesei* OEcel1B by adding copper ions into culture medium TMM + 2% cellulose. The transcription and protein level of genes under the control of *tcu1* promoter were correlated with copper availability and were inhibited severely with 20 µM copper [42]. Compared to the corresponding strains without copper, the addition of copper did not impact the cellulase production in RUT-C30, but led to a significant enhancement on cellulase production in OEcel1B (Fig. 1B). With copper added into the culture medium, the FPase, pNPCase, CMCase, pNPGase, and pNPXase activities of the strain OEcel1B were increased continuously over time, which were 25.4, 221.9, 154.7, 37.1, and 34.8 fold of OEcel1B without copper at 120 h, respectively. The protein secretion of OEcel1B with copper also increased significantly compared to that of OEcel1B without copper. At 168 h, the FPase, pNPCase, CMCase, pNPGase, and pNPXase activities for the strain OEcel1B with copper were increased to 2.45, 0.38, 0.90, 1.09, and 1.59 IU/mL, reaching 80.0%, 56.0%, 65.8%, 76.4%, and 53.8% of those in RUT-C30. This demonstrated that the deficiency of cellulase production in OEcel1B could be reversed by repressing the overexpression of *cel1b*, proving that the marked decline of cellulase activities in OEcel1B was ascribed to *cel1b* overexpression, but not its random integration into the genome.

Meanwhile, confocal observation of cell morphology showed that mycelium length of OEcel1B was only 33.2 μm, which was shorter as compared to that of RUT-C30 (98.6 μm) (Fig. 1C and Additional file 1: Fig. S4) at 168 h. More mycelium and spores were observed in RUT-C30, while there was still a large amount of cellulose existing in the culture medium of OEcel1B (Fig. 1C and Additional file 1: Fig. S4). By measuring the dry weight of the biomass using protein content [46, 47], we found OEcel1B grew at a much slower rate than that of RUT-C30 (Fig. 1D), displaying only 55.1% biomass of RUT-C30 at 168 h, which was in agreement with its very low cellulase production (Fig. 1A). The sporulation of OEcel1B was also retarded, showing only 31.0% of that of RUT-C30 on day 6 (Fig. 1E).

When cultivated on other cellulase-inducing carbon sources like cellobiose or lactose, all cellulase activities and extracellular protein concentration in strain OEcel1B were also decreased compared to that in RUT-C30, although the inhibition effect was less prominent than that on cellulose (Additional file 1: Fig. S5). The addition of copper recovered the enzyme activities and protein secretion in strains OEcel1B grown on cellobiose or lactose (Additional file 1: Fig. S5). It appears that the inhibition effect of *cel1b* overexpression on cellulase biosynthesis is universal under cellulase-producing circumstance, independent on the carbon sources.

### Overexpression of *cel1b* led to a significant decreased mRNA level of cellulase genes on cellulose

**Fig. 2:**
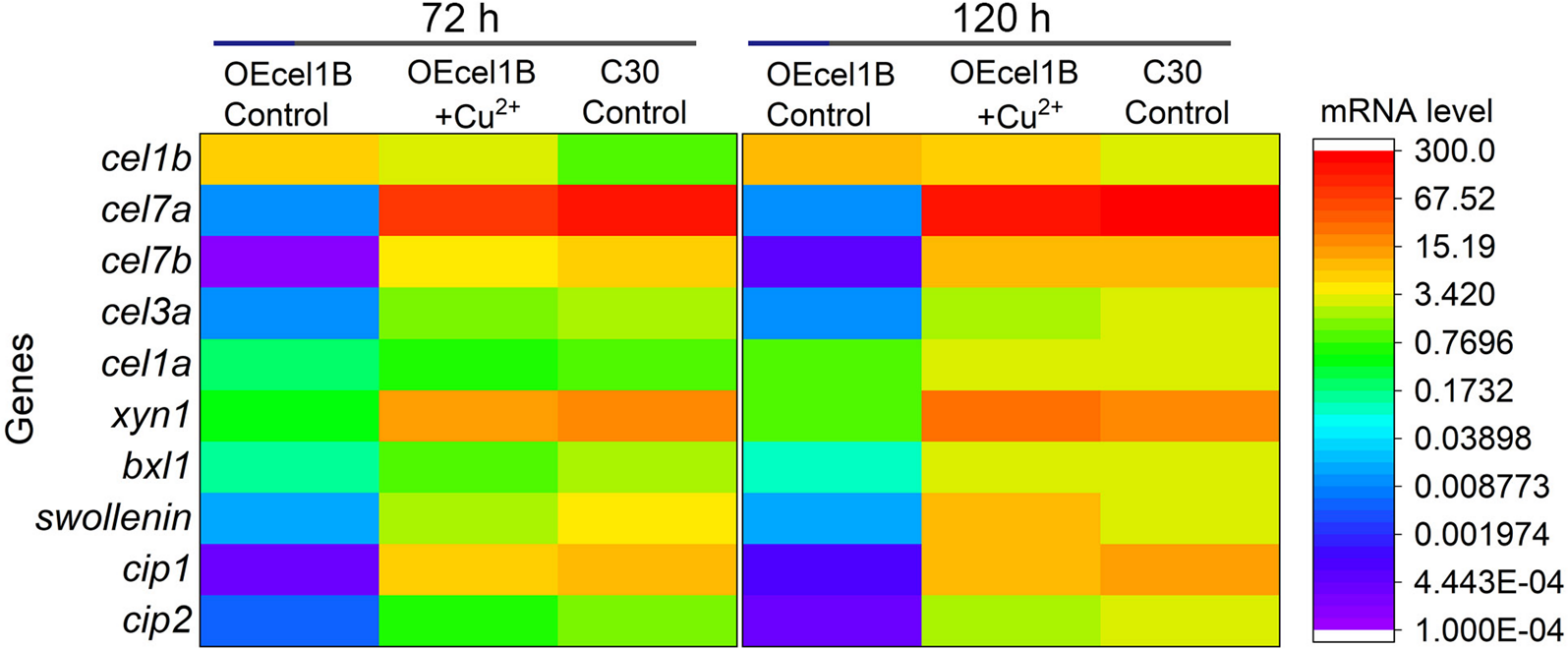
The mRNA levels of genes encoding cellulase, hemicellulase and auxiliary proteins in OEcel1B with or without Cu^2+^ and RUT-C30 cultured in TMM + 2% cellulose for 72 and 120 h

Quantitative qRT-PCR analysis was carried out to investigate how *cel1b* overexpression impacts the mRNA level of cellulase genes. In the absence of copper, only *cel1b* had an obvious increased mRNA level and all the other tested cellulase genes were downregulated in strain OEcel1B. The transcription levels of *cel1b* in OEcel1B were 4.31 and 1.71 fold higher at 72 and 120 h compared with those in RUT-C30. In our previous study, the mRNA level of *cel1b* was only 0.55 fold higher than that in RUT-C30 cultivated on cellulose at 120 h. It seems that the overexpression level of *cel1b* was not directly correlated with the promoter strength in the long-term cultivation of *T. reesei* on cellulose. Considering that *cel1b* overexpression adversely affected the growth of *T. reese*i, it was possible that the mRNA expression level of *cel1b* under *cbh1* promoter in Rcel1B was too strong to inhibit its own transcription, leading to only a slight increase on *cel1b* transcription in *T. reesei* grown on cellulose for a long term (at 120 h) compared with RUT-C30. The transcription levels of three main extracellular cellulase genes *cel7a*, *cel7b*, and *cel3a* in OEcel1B deceased notably by at least 99% compared to those in RUT-C30 at 72 h and at 120 h (Fig. 2). The hemicelllulase genes *xyn1* and *bxl1* were also repressed severely at 72 and 120 h (Fig. 2). Moreover, *swollenin*, *Cip1* and *Cip2*, which encode nonenzymatic cellulose attacking enzymes to promote cellulose degradation, were also downregulated significantly (Fig. 2). Among the tested cellulase genes, β-glucosidase *cel1a* was the least affected gene, whose mRNA level was decreased by 74.4% at 72 h. The decreased transcription level of cellulase genes was in good accordance with decreased enzyme production and protein secretion as shown in Fig. 1.

With copper added into the culture medium, *cel1b* in OEcel1B was repressed sharply, whose expression was only about half of that in OEcel1B without copper and only 2.38 fold that in RUT-C30 at 72 h. In contrast, the mRNA levels of all the other tested cellulase genes in OEcel1B were increased with the treatment of copper, which was recovered to 40.6–75.1% of those in RUT-C30. The inhibition effects of *cel7b* and *bxl1* were completely relieved at 120 h, reaching a comparable level of those in RUT-C30. The mRNA levels of *cel7a*, *cel3a*, *cel1a*, *cip1*, and *cip2* were also increased to 66.1–87.2% of those in RUT-C30. Interestingly, *xyn1* and *swollenin* were upregulated by 34.4% and 128.25% than those in RUT-C30. All these demonstrated that the addition of copper inhibited the overexpression of *cel1b* and de-repressed the cellulase genes, contributing to the recovery of enzyme activities in OEcel1B (Fig. 1B).

### The deletion of *cel1b* slightly influenced cellulase production

**Fig. 3:**
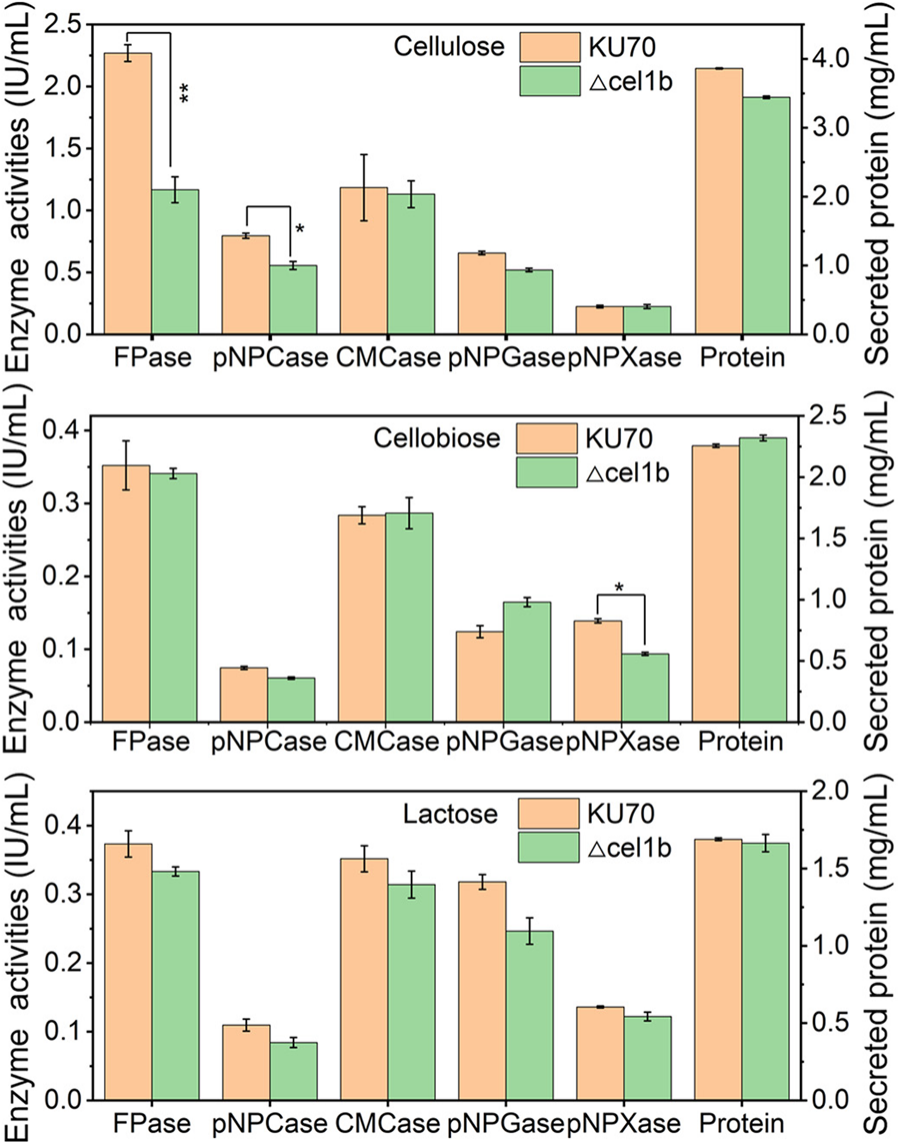
Cellulase activities and protein secretion for *T. reesei* KU70 and △cel1b at 120 h on TMM induced by 2% cellulose, 1% cellobiose, and 2% lactose, respectively. Data are represented as the mean of three independent experiments, and error bars express the standard deviations. Asterisks indicate significant differences (*p < 0.05, **p < 0.01, ***p < 0.001) as assessed by Student’s *t* test

To further explore the function of *cel1b* in cellulase biosynthesis, gene *cel1b* was knockout in *T. reesei* KU70, yielding the mutant strain △cel1b (Additional file 1: Fig. S2C). Except a decline by 48.6% and 30.0% on FPase and pNPCase activity respectively, no significant differences were observed on CMCase, pNPGase, and pNPXase activity and secreted protein between △cel1b and KU70 at 120 h grown on cellulose (Fig. 3). Meanwhile, the mRNA levels of genes involved in cellulase production, including cellulase genes, transcriptional factors, and sugar transporters at 72 h were measured by qRT-PCR and no significant change was observed in these genes between KU70 and △cel1b (Additional file 1: Fig. S6). This result is consistent with that the deletion of *cel1b* did not markedly influence the cellulase activities. When culturing on cellobiose or lactose, the cellulase activities and protein secretion of strain △cel1b was not markedly changed as compared to KU70. Particularly, the pNPGase activity of △cel1b was increased by 32.6% when using cellobiose as the only carbon source. In contrast to a significant inhibition of *cel1b* overexpression on cellulase production, the deletion of *cel1b* did not noticeably affect the cellulase production, which might be ascribed to the existence of functional equivalent β-glucosidase *cel1a* [33].

The effects of *cel1b* deletion on cell growth, spore ability, and morphology of *T. reesei* were also evaluated under cellulase-producing condition (Additional file 1: Fig. S7). The colony diameter and spore amount of strain △cel1b were similar to that of the parental strain KU70. The confocal imaging showed that the hyphae morphology of △cel1b was not changed significantly as compared to KU70. Taken together, the deletion of *cel1b* in *T. reesei* did not markedly influence the phenotype of *T. reesei* including cell growth, spore ability, and morphology.

### Cellular distribution of CEL1B

**Fig. 4:**
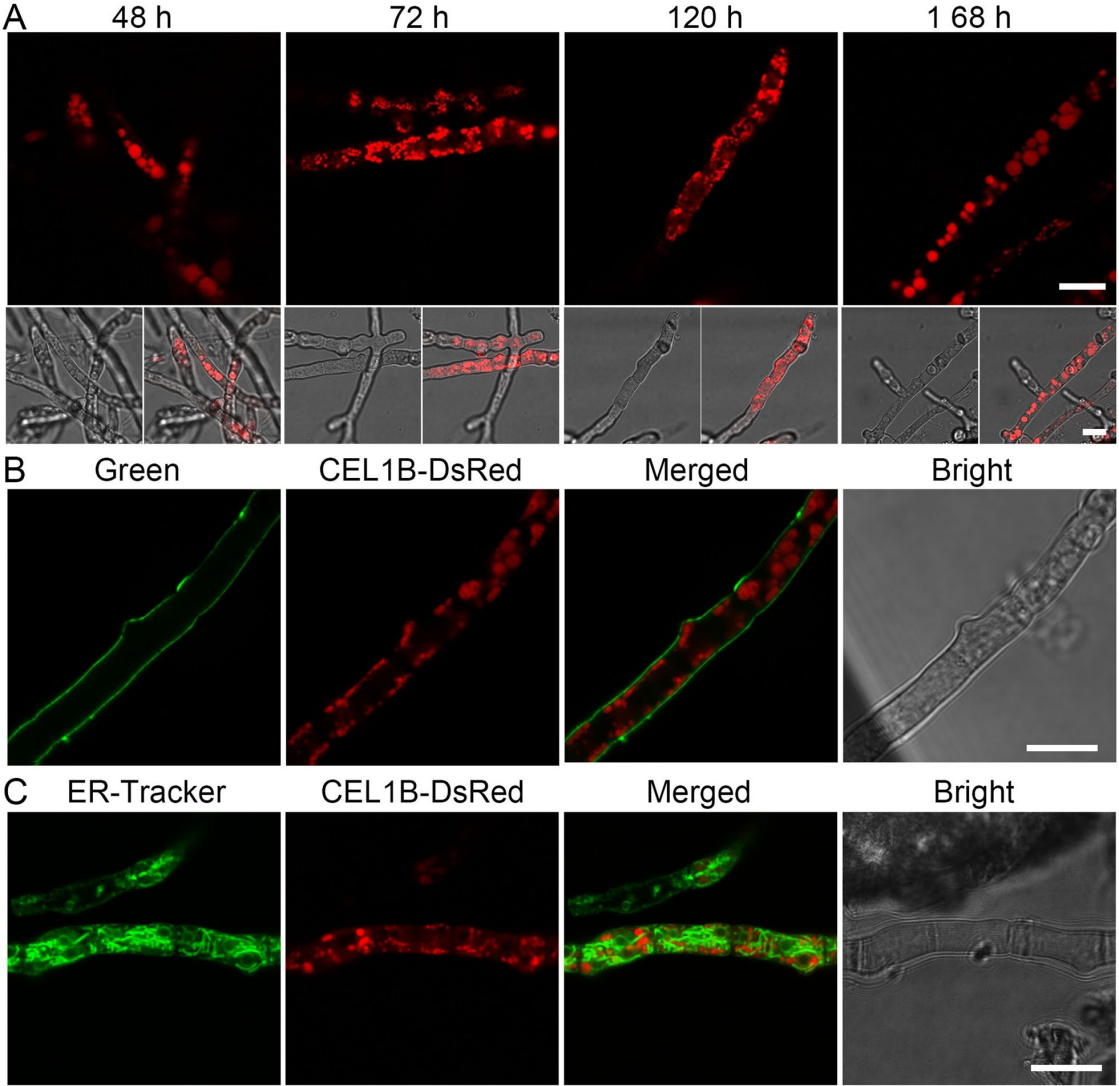
Confocal images of CEL1B-DsRed in strain OEcel1B cultured on TMM + 2% cellulose. **A** Cellular distribution of CEL1B-DsRed. **B**, **C** Confocal images of strain OEcel1B stained with GC-PEG-cholesterol-FITC, a green fluorescence dye for cell wall (**B**) or ER-Tracker (**C**) at 168 h. Scale bar = 10 μm

To investigate its cellular distribution, the C terminus of CEL1B were fused with red fluorescent protein (DsRed) as reported previously (Additional file 1: Fig. S2) [22]. Among the three *cel1b*-overexpressing strains cultivated in TMM + 2% cellulose, the cellular distribution of CEL1B-DsRed could only be observed in strain OEcel1B during 7 days’ fermentation process by confocal laser scanning microscopy (CLSM) (Fig. 4), while the red fluorescence in strains Rcel1B and OEcel1B-P_*bxl1*_ could not be observed. Red fluorescence appeared at 48 h in OEcel1B and two types of cellular distribution patterns of CEL1B-DsRed were found (Fig. 4A). Some fluorescence was located in vacuoles, while some was diffused along the cell membrane/wall with protein aggregation distributing in the cytoplasm (Fig. 4A). A green fluorescent dye GC-PEG-cholesterol-FITC for cell wall imaging was employed to stain OEcel1B [48], but no yellow fluorescence in the merged figures was found (Fig. 4B), indicating that CEL1B was distributed on cell membrane, but not cell wall. Using ER-Tracker with green fluorescence to stain OEcel1B, a little yellow fluorescence was observed (Fig. 4C), indicating CEL1B-DsRed might reside in ER. However, the yellow fluorescence was not that significant and more evidence is required for confirmation. The fluorescence intensity of supernatant in OEcel1B almost stayed unchanged during the whole fermentation process, similar to that of RUT-C30, suggesting that CEL1B was not secreted into the culture medium (Additional file 1: Fig. S8), in agreement with a previous study that CEL1B was an intracellular β-glucosidase [30]. When using 2% lactose or 1% cellobiose as the carbon source, the red fluorescence was similar to that observed on cellulose, which implied that the cellular distribution of CEL1B was independent on carbon sources (Additional file 1: Fig. S9 and S10).

### The cellulase inhibition by *cel1b* overexpression was not due to β-glucosidase function

**Fig. 5:**
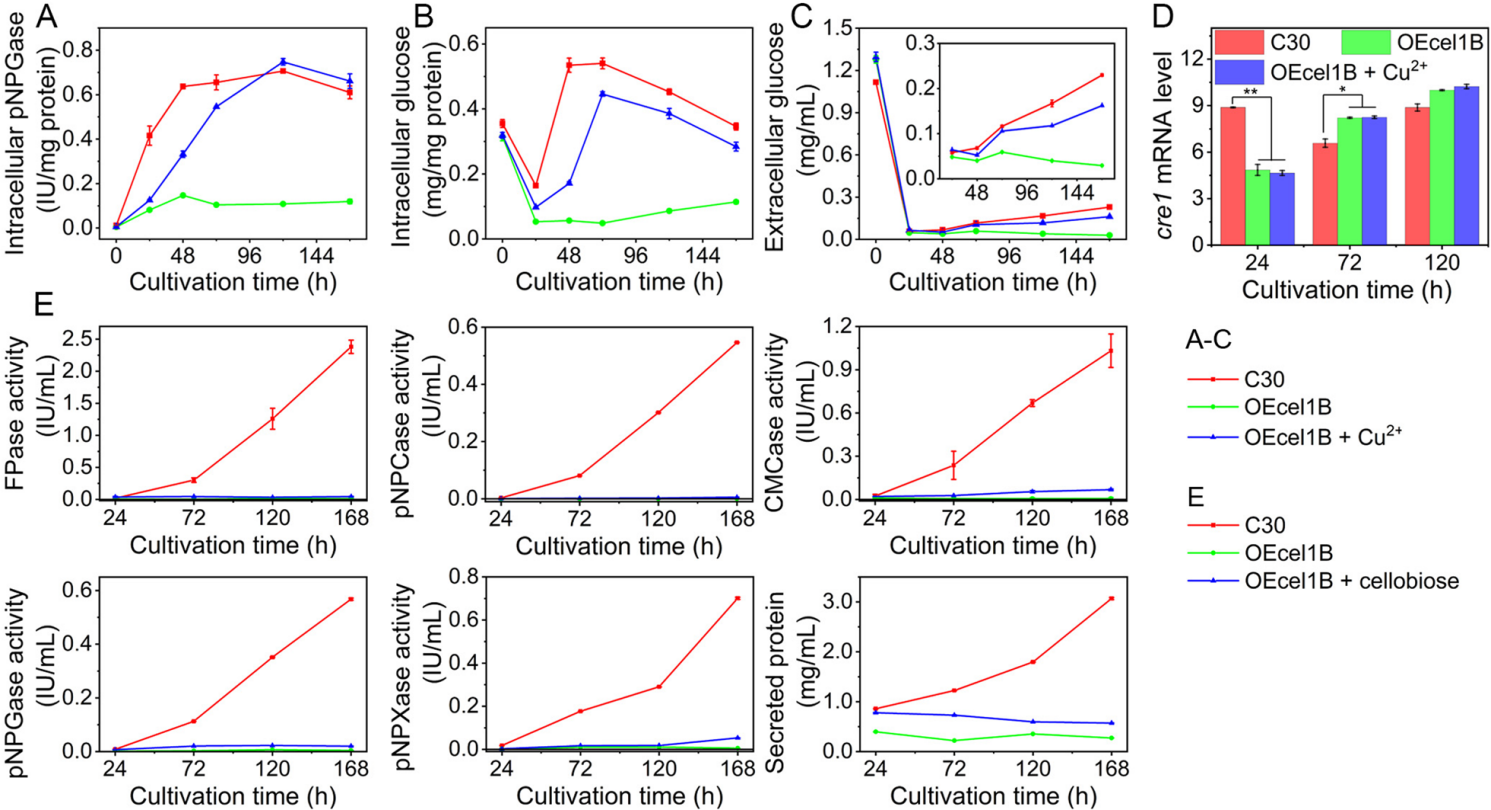
**A** Intracellular pNPGase activity. **B**, **C** Intracellular and extracellular glucose concentration. **D** The relative mRNA level of *cre1*. **E** Cellulase activities and protein secretion of recombinant *T. reesei* OEcel1B with/without 0.25% cellobiose. Strains OEcel1B with/without Cu^2+^ and RUT-C30 were cultured in TMM + 2% cellulose for 168 h. Data are represented as the mean of three independent experiments, and error bars express the standard deviations. Asterisks indicate significant differences (*p < 0.05, **p < 0.01, ***p < 0.001) as assessed by Student’s *t* test

Since CEL1B was identified to be intracellular, it might work as an intracellular β-glucosidase responsible for hydrolyzing disaccharide released by EG and CBH to two molecules of glucose [32]. Therefore, the *cel1b* overexpression might produce more intracellular β-glucosidase to convert more cellobiose to more glucose, causing the deficiency of intracellular cellobiose (a well-known inducer for cellulase), and increased intracellular glucose that subsequently activate carbon catabolite repression (CCR), both of which can inhibit cellulase production. To test this hypothesis, we measured the intracellular pNPGase activity, both extracellular and intracellular glucose concentration, and the transcriptional level of carbon catabolite repressor *cre1* in *T. reesei*. The intracellular pNPGase activity in RUT-C30 was increased at the first 48 h to reach a plateau phase from 48 to 144 h. Surprisingly, a dramatic decline of intracellular pNPGase activity was observed in strain OEcel1B by comparing to RUT-C30 (Fig. 5A). Consistent with this reduction of intracellular pNPGase activity, the intracellular glucose concentration in OEcel1B was much lower than that of RUT-C30 during the whole fermentation process (Fig. 5B). The extracellular glucose concentration of OEcel1B and RUT-C30 were all decreased in the culture supernatant at the first 24 h to about 0.05 mg/mL (Fig. 5C). Then the extracellular glucose concentration of RUT-C30 kept increasing during the rest fermentation to 0.23 mg/mL at 168 h, while the extracellular glucose concentration of OEcel1B was decreased continuously to 0.03 mg/mL at 168 h, much lower than that of RUT-C30 (Fig. 5C). Meanwhile, the mRNA level of *cre1* in OEcel1B was reduced significantly at 24 h, but did not have a significant change at 72 and 120 h, as compared to that in RUT-C30 (Fig. 5D). When copper was added into the culture medium to inhibit the *cel1b* overexpression in OEcel1B, the intracellular pNPGase activity, and the extracellular and intracellular glucose in OEcel1B were all increased remarkably, showing a similar trend to that of RUT-C30.

We were unable to detect intracellular or extracellular cellobiose in *T. reesei* grown on cellulose by high-performance liquid chromatography (HPLC) due to that the level was too low to be detected. Instead, 0.25% cellobiose was added into TMM + 2% cellulose to see whether the cellulase inhibition by *cel1b* overexpression was caused by cellobiose deficiency. All (hemi)cellulase activities and secreted protein concentration of OEcel1B with the supply of cellobiose had a very limited increase compared to that of OEcel1B without cellobiose and remained to be much less than that of RUT-C30 (Fig. 5E), implicating that the cellulase inhibition might not be owing to cellobiose deficiency. Overall, the overexpression of *cel1b* led to the reduction of intracellular pNPGase activity and glucose concentration in cells without inducing CCR, while the cellobiose addition could not restore the cellulase repression induced by *cel1b* overexpression. It seems that the cellulase inhibition effect by *cel1b* overexpression was not due to the function of *cel1b* as an intracellular β-glucosidase.

### The *cel1b* overexpression impacted significantly the transmembrane transport process and endoplasmic reticulum function

**Fig. 6:**
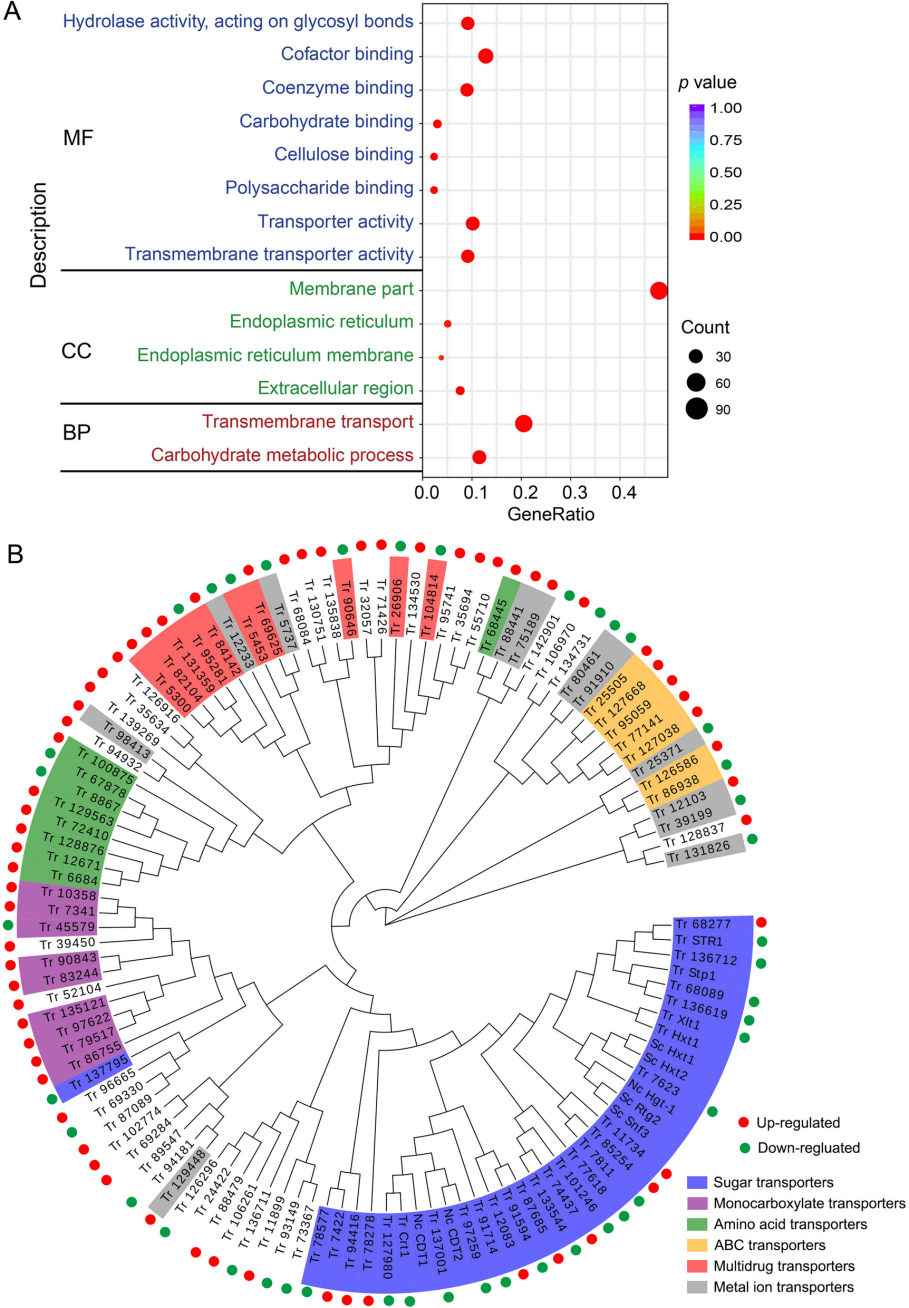
**A** Gene ontology (GO) functional enrichment analysis of DEGs. The y axis represents the name of the most enriched GOs that belong to different ontologies. MF: the molecular function; CC: the cellular component; BP: the biological process; GeneRatio: the number of DEGs in a specified GO term/ the number of the total DEGs in all GO terms; Count: the number of DEGs in a specified GO term. **B** Phylogenetic analysis of DEGs in the “transmembrane transport” process. The neighbor-joining tree was generated with Mega 5 and EvolView. Tr: *Trichoderma reesei*; Sc: *Saccharomyces cerevisiae*; Nc: *Neurospora crassa*

RNA-seq analysis was performed to explore the mechanism underlying the inhibition of *cel1b* overexpression on the cellulase activity in *T. reesei* at the transcriptional level. Of the 10,553 genes present in the genome of *T. reesei*, 1795 were differentially expressed in the strain OEcel1B as compared to RUT-C30 cultivated on cellulose (Additional file 2: Table S1). Among these, 1064 were upregulated and 731 were downregulated. Gene ontology (GO) functional enrichment analysis of these DEGs showed that the most enriched biological processes (BP) were “carbohydrate metabolic process” and “transmembrane transport” which included 60 and 108 genes respectively (Fig. 6A). 76.7% DEGs related to “carbohydrate metabolic process” were significantly downregulated (Additional file 3: Table S2), matching well with the declined cellulase activities (Fig. 1). As the enriched cellular components (CC) shown, these DEGs were majorly localized in membrane, extracellular region, and endoplasmic reticulum (ER). For the enriched molecular function (MF), the most enriched DEGs belong to “hydrolase activity”, “cofactor binding”, and “transporter activity”. The molecular function “cellulose binding” was also enriched. All these results demonstrated that the *cel1b* overexpression influenced significantly the transmembrane transport process and ER function in addition to the cellulase inhibition.

For the “transmembrane transport” process, 66 genes were upregulated and 42 were downregulated, among which major facilitator superfamily (MFS) transporters were the most abundant (Additional file 4: Table S3). MFS transporters are essential for the movement of various substances such as nutrients, metabolites, signaling molecules, toxins and drugs across biological membranes [49]. To better understand the function of these DEGs involved in the “transmembrane transport” process, phylogenetic analysis was carried out using their amino acid sequences (Fig. 6B). Six transporter families including sugar transporters, monocarboxylate transporters, amino acid transporters, ABC transporters, multidrug transporters, and metal ion transporters were affected by the overexpression of *cel1b*. The largest cluster was formed by sugar transporters containing 27 DEGs. The well-known cellulase-activating sugar transporter Crt1 (M419DRAFT_109243) transporting lactose [50] and cellobiose [51] was downregulated together with M419DRAFT_91594 transporting cellobiose, xylose, and mannose [52]. The most repressed sugar transporter in strain OEcel1B was hexose transporter (M419DRAFT_97259) that is similar to CDT2 in *N. crassa*, only 0.73% of that in RUT-C30 at mRNA level (Additional file 4: Table S3). Another putative glucose transporter M419DRAFT_7623, which is similar to Hxt1/2 in *S. cerevisiae*, was also downregulated. The lactose permeases (M419DRAFT_137795, M419DRAFT_127980, and M419DRAFT_137001) [29], and xylose transporters Str1 (M419DRAFT_138519) [53, 54] and Xlt1 (M419DRAFT_33630) [55] were all repressed to different degrees. Lactose permease M419DRAFT_127980 is similar to Crt1, and both are homologues to CDT1 in *N. crassa.*

To test whether the cell wall integrity was influenced by the overexpression of *cel1b*, strains RUT-C30 and OEcel1B were treated with different concentrations of Congo red (CR) and NaCl (Additional file 1: Fig. S11). The colony diameter of OEcel1B was only 52.5% or 58.5% that of RUT-C30 in the presence of 75 µg/mL CR or 0.6 mol/L NaCl (Additional file 1: Fig. S11), illustrating that the overexpression of *cel1b* made *T. reesei* more sensitive to cell wall interfering agents.

### The cellular uptake of sugar was hampered with the overexpression of *cel1b*

**Fig. 7:**
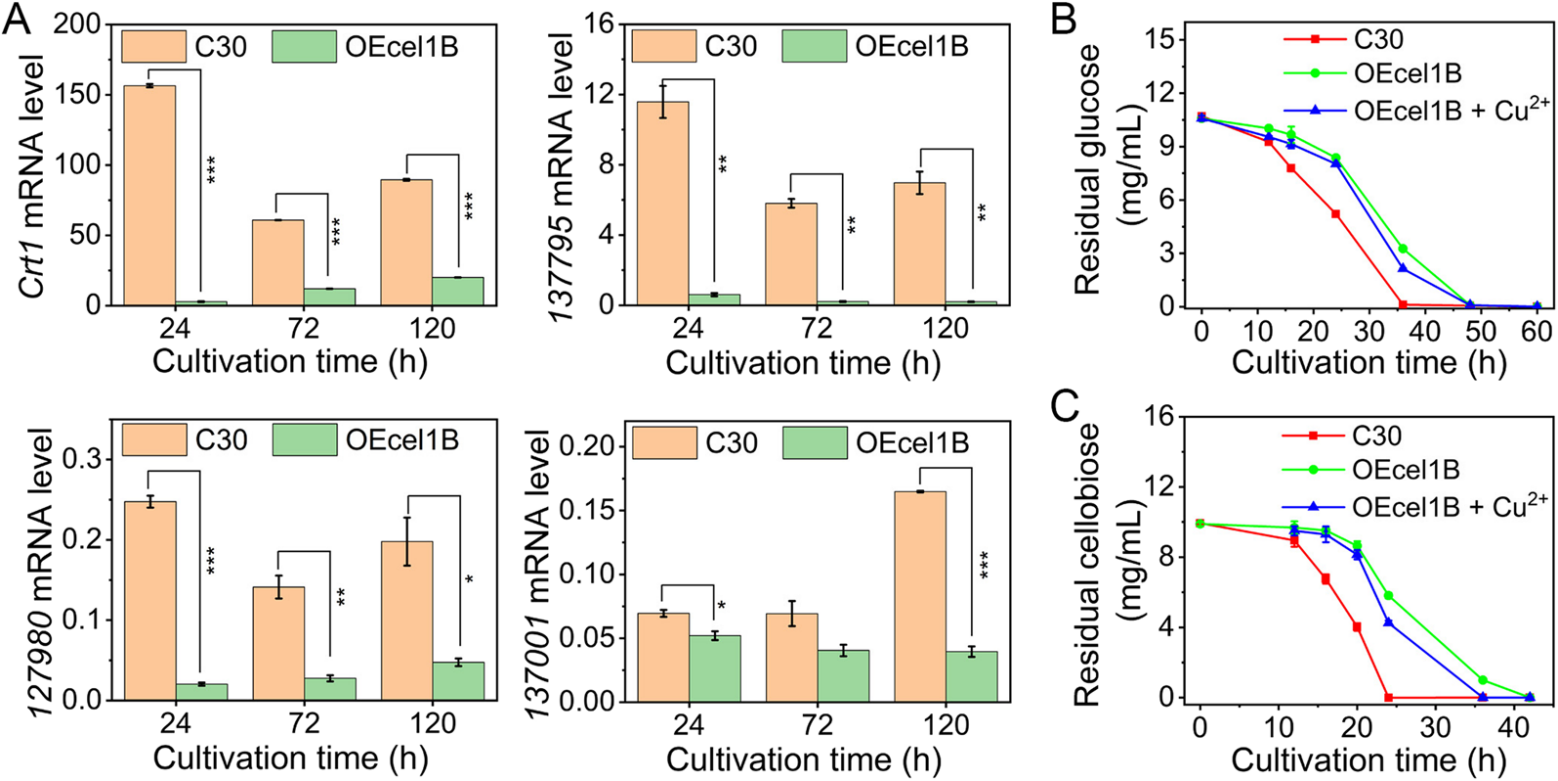
**A** qRT-PCR analysis of genes encoding sugar transporters. Strains OEcel1B and RUT-C30 were cultured in TMM + 2% cellulose for 120 h. **B**, **C** The residual glucose (**B**) and cellobiose (**C**) in the supernatant of *T. reesei* RUT-C30 and OEcel1B, which were cultured in TMM using 1% glucose or cellobiose as the sole carbon source, respectively. Asterisks indicate significant differences (*p < 0.05, **p < 0.01, ***p < 0.001) as assessed by Student’s *t* test

To further evidence the transcriptome result, the mRNA levels of sugar transporters in RUT-C30 and OEcel1B cultivated on cellulose at different time points were further analyzed by qRT-PCR. The mRNA levels of Crt1, M419DRAFT_137795, M419DRAFT_127980, and M419DRAFT_137001 kept much lower than those in RUT-C30 during the whole fermentation period (Fig. 7A), which was consistent with the transcriptome result of these sugar transporters. Inspired by the significant expression change of sugar transporter in OEcel1B, we assayed the sugar uptake of RUT-C30 and OEcel1B cells by measuring the residual sugar in the culture supernatant using glucose or cellobiose as the sole carbon source. The cellular uptake rate of glucose or cellobiose for OEcel1B was much slower than that for RUT-C30, as indicated by that the residual glucose or cellobiose concentration was higher for OEcel1B than for RUT-C30. It took 48 and 42 h for OEcel1B to exhaust the glucose and cellobiose in TMM culture medium respectively, longer than that for RUT-C30 (36 and 24 h) (Fig. 7B). Collectively, the overexpression of *cel1b* downregulated the transcriptional levels of vital sugar transporters, leading to the retarded cellular uptake of sugars such as glucose and cellobiose.

### The overexpression of *cel1b* adversely affects the function of ER without causing ER stress

**Fig. 8:**
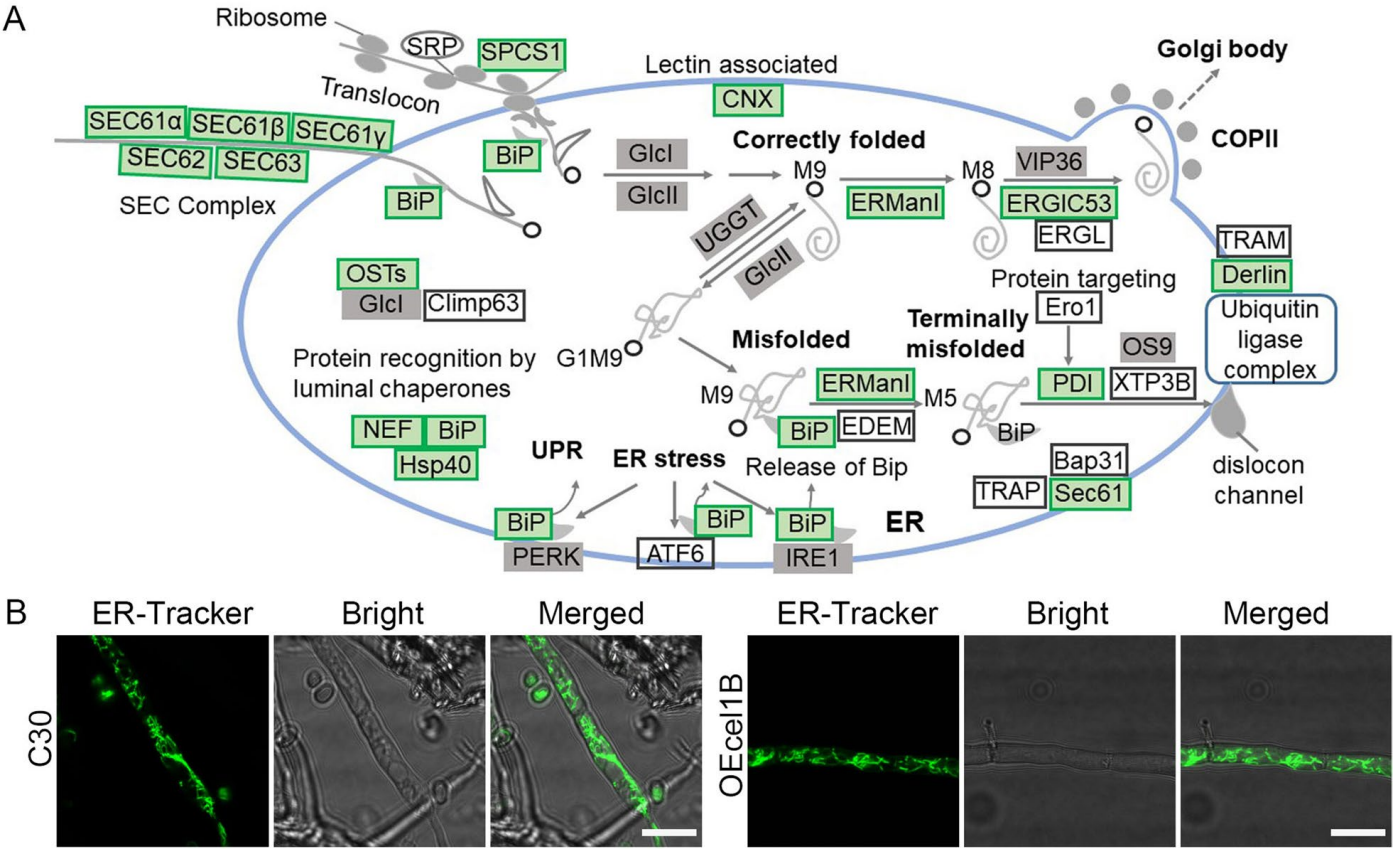
**A** Genes involved in ER function. The downregulated and upregulated DEGs were shown in green and red rectangles respectively, while genes that was identified in *T. reesei* but not differentially expressed in strain OEcel1B was presented in gray rectangles. **B** Confocal images of strains RUT-C30 and OEcel1B stained with the green emissive ER-Tracker at 168 h. Scale bar = 10 μm

The category “Protein export” and “Protein processing in endoplasmic reticulum” were among the enriched KEGG pathways (Additional file 1: Fig. S12), in line with the enriched category “Endoplasmic reticulum” for cellular components (Fig. 6A). 7 genes involved in protein translocation across ER were downregulated significantly (Additional file 5: Table S4), including the components of the SEC complex (SEC61α, SEC61β, SEC61γ, SEC62, and SEC63) and ER chaperone BiP1 that are essential for post-translational translocation of protein into ER [56], and signal peptidase complex subunit 1 SPCS1 related to co-translational translocation of protein to ER (Fig. 8A). SPCS1, a component of the microsomal signal peptidase complex, is responsible for cleaving signal peptides of secreted and membrane-related proteins [57]. BiP1, an important ER-chaperone functioning in the various processes, is required for protein translocation across the membrane of ER, folding nascent proteins into their native state with HSP40 and nucleotide exchange factors (NEF), degradation of misfolded proteins, and the unfolded protein response (UPR) regulation (Fig. 8A) [58, 59]. ER mannosidase I (ERManI, M419DRAFT_83445 and M419DRAFT_101105) responsible for trimming of nascent glycoprotein *N*-glycans, the lectin chaperone calnexin (CNX, M419DRAFT_135283), and protein disulfide isomerase (PDI, M419DRAFT_111778) were declined at the transcriptional level. Interestingly, except the markedly downregulated BiP1 and PDI, no DEG was related to ER stress. For instance, the vital ER stress sensors of UPR including PERK, IRE1, and ATF6 were not found in DEGs. Moreover, when the green-emitting ER-tracker was utilized to stain ER in strains RUT-C30 and OEcel1B, there was no difference of ER morphology between OEcel1B and RUT-C30 (Fig. 8B). It seems that the *cel1b* overexpression does not cause burden to ER and the pathways related to ER stress were not activated, which might be reasonable given that we did not observe obvious residence of CEL1B-DsRed in ER. All these implied that *cel1b* overexpression blocked protein translocation into ER and jeopardized the protein processing in ER without inducing ER stress, of which both hold keys to proper folding, maturation and secretion of cellulase.

## Discussion

**Fig. 9:**
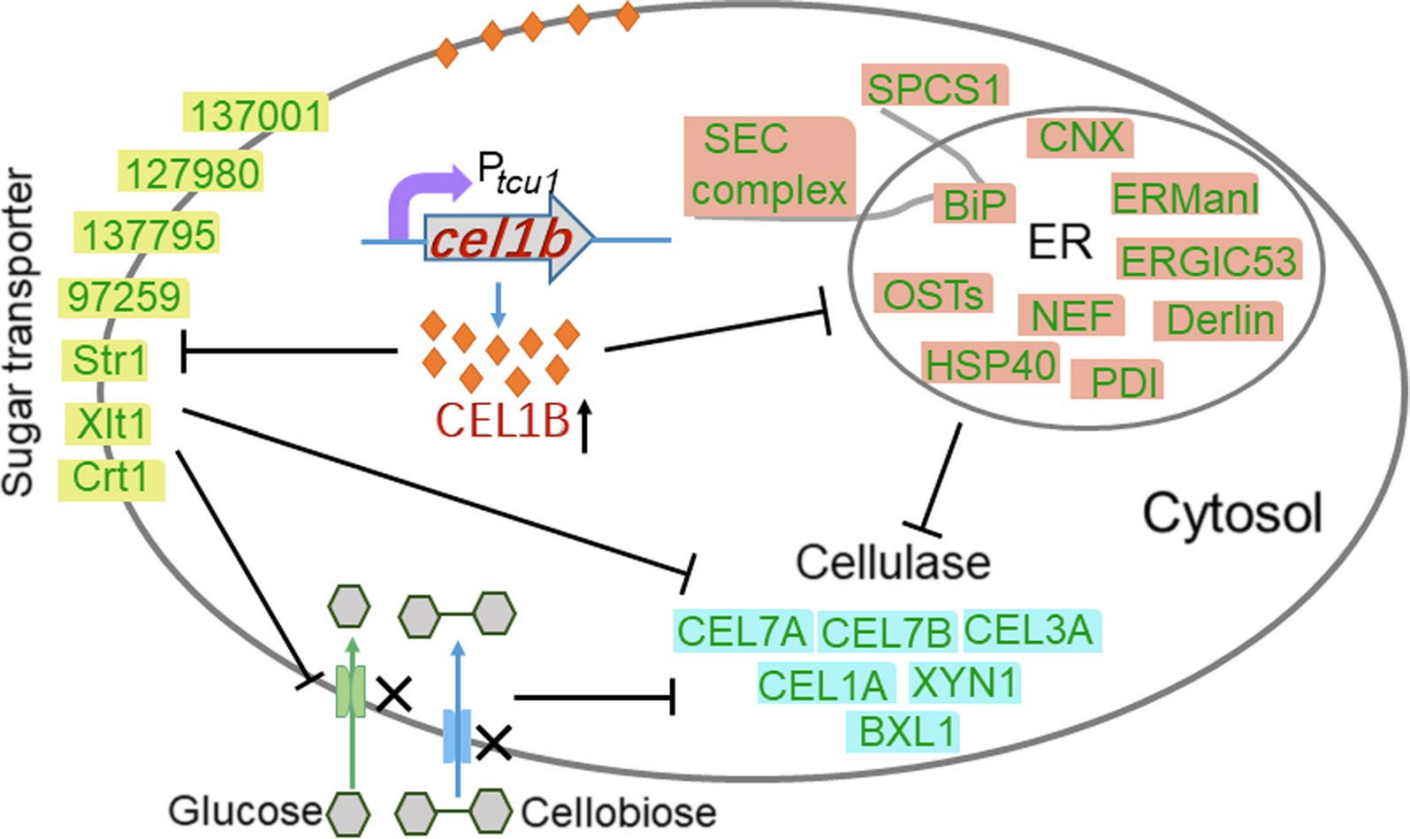
A proposed model of cellulase inhibition process induced by *cel1b* overexpression in *T. reesei*

Gene *cel1b* is predicted to possess the binding sites for several transcriptional factors relevant to cellulase biosynthesis [34, 36–39] and the mRNA level of *cel1b* was reported to be related with the cellulase production [33–35], demonstrating that *cel1b* is of great importance for cellulase production in filamentous fungi. Our previous study showed that among 11 β-glucosidases predicted in *T. reesei*, only putative β-glucosidase *cel1b* overexpression almost completely abolished cellulase production in *T. reesei* [22], which triggered our great interest. Therefore, in this study, we further evidenced that the cellulase inhibition was indeed caused by the overexpression of *cel1b* and explored extensively the mechanisms behind this phenomenon to reveal the role of *cel1b* in cellulase production in *T. reesei* (Fig. 9). The dramatic decrease of cellulase biosynthesis in *T. reesei* mutants OEcel1B and OEcel1B-P_*bxl1*_ overexpressing β-glucosidase *cel1b* under promoters *tcu1* and *bxl1* respectively, confirmed our previous result that the *cel1b* overexpression under promoter *cbh1* abolished cellulase production [22]. Taking advantage of copper-repressed *tcu1* promoter, the addition of copper into the culture medium retarded the *cel1b* overexpression and restored the cellulase production in OEcel1B, demonstrating that the cellulase inhibition in strain OEcel1B was indeed due to the overexpression of *cel1b*, but not the random insertion of the overexpression cassette. The inhibition effect was also observed at the transcriptional levels of cellulase-related genes, which was relieved when the mRNA level of *cel1b* in OEcel1B was reduced with the treatment of copper ions. Nevertheless, the cellulase production was not hampered when overexpressing other β-glucosidases in *T. reesei* [22, 26, 28], making *cel1b* very special.

CEL1B was further evidenced as intracellular in this study, giving the possibility that it might act as an intracellular β-glucosidase for converting cellobiose to glucose to increase intracellular glucose level and decrease intracellular cellobiose level, leading to the cellulase inhibition. However, our data showed the overexpression of *cel1b* reduced pNPGase activity and glucose concentration in OEcel1B cells, while the cellobiose addition at low concentration could not restore the cellulase repression, implying that the cellulase repression was not due to the function of CEL1B as an intracellular β-glucosidase. This is reasonable given that CEL1B exhibited the lowest substrate specific activity toward pNPG, G3, G4, and Gen compared to other β-glucosidases in *T. reesei* [30]. It seems that CEL1B might not function as intracellular β-glucosidase in *T. reesei*.

On the other hand, transcriptome data showed that the enriched DEGs in OEcel1B compared with RUT-C30 were related to transmembrane transport process and ER function as well as cellulase biosynthesis. Transporter families such as sugar transporters, monocarboxylate transporters, amino acid transporters, ABC transporters, multidrug transporters, and metal ion transporters were influenced by the overexpression of *cel1b* with sugar transporters forming the largest cluster as shown by phylogenetic analysis. Some vital transmembrane transporters transporting glucose, cellobiose, and lactose were significantly downregulated, as confirmed by qRT-PCR (Fig. 7A). As a result, the much slower cellular uptake of glucose and cellobiose in OEcel1B was observed than that in RUT-C30 (Fig. 7B). Also, OEcel1B displayed higher sensitivity to cell wall interfering compounds CR and NaCl (Additional file 1: Fig. S11). Collectively, these results point to that the overexpression of *cel1b* exerts a marked influence on the transmembrane process, particularly the cellular entry of sugar, which might contribute to the cellulase repression under *cel1b* overexpression. This is sensible considering that *cel1b* was localized on the cell membrane (Fig. 4). Moreover, when using cellobiose or lactose as the carbon source for cellulase production, the inhibition impact of the *cel1b* overexpression was partly alleviated in OEcel1B (Additional file 1: Fig. S5), further supporting that the impaired sugar transport induced by overexpressing *cel1b* contributed to the cellulase inhibition.

Previous studies have shown that sugar transporters are of great importance for (hemi)cellulase production in filamentous fungi. The absence of some downregulated sugar transporters found here have been reported to reduce cellulase production, such as Crt1 [50, 51], M419DRAFT_97259, M419DRAFT_137001 [29], M419DRAFT_137795 [29], and Str1 [53, 54]. For instance, deletion of sugar transporter Crt1 in *T. reesei* QM9414 or TU_6 caused a severe defect on growth and cellulase induction on lactose or Avicel, which was shown to be essential for cellulase production [50, 60]. The double knockout of CDT1 and CDT2 in *N. crassa*, the homologues of sugar transporter Crt1 and hexose transporter M419DRAFT_97259 respectively, made *N. crassa* unable to induce cellulase gene expression in response to crystalline cellulose [61]. Surprisingly, the putative hexose transporter M419DRAFT_7623 similar to Hxt1/2 in *S. cerevisiae* was significantly declined, although it has been reported that the deletion of glucose transporters such as *hgt-1* and *hgt-2* in *N. crassa*, and *Stp1* in *T. reesei* can improve the cellulase production [60, 62]. Meanwhile, the effects of β-glucosidases on sugar transporter have been reported in a previous research. The knockout of β-glucosidase *cel3g* can upregulate the transcription of lactose permease Crt1, M419DRAFT_137795, and M419DRAFT_137001 that might be involved in lactose transport [29], leading to a significant improvement of cellulase production in *T. reesei* mutant strain △*bgl3i*. Interestingly, *cel1b* locates next to the positive transcriptional factor Ace3 for cellulase and lactose transporter M419DRAFT_137001 [63], of which both were downregulated under *cel1b* overexpression. These previous results provide more evidences to support our conclusion that *cel1b* inhibits cellulase biosynthesis through affecting sugar transporters.

Another interesting thing is that the overexpression of *cel1b* affected ER function. ER is an important cellular compartment responsible for synthesis and folding of newly synthesized proteins destined for secretion [64]. Protein transport into ER can occur either co- or post-translationally which required different components [65]. In yeast, the Sec61 channel cooperates with the hetero-tetrameric Sec63 complex (Sec62, Sec63, Sec71, and Sec72), which was called the Sec machinery that is essential for post-translational import into the ER. During co-translational transport, signal sequences are recognized by the signal recognition particle (SRP) and are targeted to the Sec machinery. Obviously, the overexpression of *cel1b* probably made both strategies inoperative as shown by the decreased mRNA levels of Sec61 complex, Sec63 complex, BiP, and SPCS1, eliciting an inefficient protein translocation into ER. Meanwhile, the genes involved in protein processing like CNX, ERManI, and PDI were decreased. Meanwhile, despite the notably decreased expression of BiP1 and PDI, the expression of genes associated with ER stress were not significantly changed. In addition, the size and shape of ER did not have a significant difference by *cel1b* overexpression (Fig. 8). These findings indicate that the overexpression of *cel1b* did not trigger ER stress, which is reasonable given that most of the overexpressed CEL1B was not localized in ER (Fig. 4). All these demonstrated that overexpression of *cel1b* did not trigger ER stress and UPR activation, but inhibited the ER function, which resulted in a dramatic decline on protein secretion into the extracellular space. The previous study has shown that cellulase secretion was majorly through the classic ER-Golgi secretory pathway [66]. The super secretion ability of *T. reesei* RUT-C30 was attributed to its highly abundant ER [67]. Thus, the dysfunction of ER in protein export and protein processing might be responsible for the cellulase repression under the *cel1b* overexpression.

In summary, we reported that the predicted β-glucosidase CEL1B probably did not act as β-glucosidase in *T. reesei*. Instead, its overexpression caused cellulase repression by damaging the function of sugar transport and ER (Fig. 9). The overexpression of *cel1b* driven by *cbh1*, *tcu1*, and *bxl1* promoters in *T. reesei* RUT-C30 dramatically decreased cellulase production regardless of carbon sources. In agreement with the reduced cellulase production, the mRNA levels of genes related to the cellulase production were decreased severely except *cel1b*. The *cel1b* overexpression also hampered cell growth and sporulation, and shortened the fungal hyphae. In contrast, the deletion of *cel1b* does not noticeably impact cellulase production. Protein CEL1B was shown to be intracellular, being located in vacuole and cell membrane. The mechanism investigation demonstrated that the cellulase inhibition in *cel1b-*overexpressing strain OEcel1B was not due to β-glucosidase activity, for the *cel1b* overexpression decreased the intracellular pNPGase activity and intracellular/extracellular glucose concentration without causing carbon catabolite repression. On the other hand, RNA-sequencing analysis showed the transmembrane transport process and ER function were affected noticeably by overexpressing *cel1b*. Genes encoding some vital sugar transporters like Ctr1, M419DRAFT_137795, M419DRAFT_127980, and M419DRAFT_137001 were noticeably decreased and the cellular uptake of sugar including glucose and cellobiose was compromised severely. This dysfunction of the transmembrane transport process and ER might be associated with the inhibition of cellulase production. All these results will be beneficial for delineating the entire regulation mechanism of fungal cellulase synthesis and rationally enhancing cellulase production in industry.

## Methods

### Microorganisms, plasmids and cultivation conditions

*Trichoderma reesei* RUT-C30 (CICC 13052, ATCC 56765) was purchased from China Center of Industrial Culture Collection. *T. reesei* KU70 and the plasmid pXBthg were provided friendly by Professor Wei Wang and Zhihua Zhou. *Escherichia coli* DH5α was used as the cloning host for plasmid construction. *T. reesei* were grown on potato dextrose agar (PDA) plates for conidia production and in *Trichoderma* minimal media (TMM) [2] with 2% (w/t) cellulose or other carbon sources as indicated at 28 ℃ with 220 rpm. *Agrobacterium tumefaciens* AGL-1 was used as a T-DNA donor for fungal transformation. *E. coli* DH5α and *A. tumefaciens* AGL-1 were cultivated in Luria-Bertani (LB) with 220 rpm at 37 ℃ and 28 ℃, respectively. All chemicals used in this research were ordered from Sigma-Aldrich, USA.

### Shake flask cultivation

5% (v/v, 10^7^/mL) conidia of *T. reesei* were cultured into 10 mL sabouraud dextrose broth (SDB) at 28 ℃ with 200 rpm for 2 days. 5 mL pre-grown mycelia were inoculated into 50 mL TMM media (pH 6) plus 2% cellulose or other carbon sources as indicated in the text with/without 20 µM CuSO_4_, and incubated at 28 ℃ for 7 days. Samples were taken at different time points, and if required centrifuged at 8000 rpm for 20 min. The supernatants were utilized for (hemi)cellulase activity, residual glucose/cellobiose assay, and fluorescence intensity detection, while the mycelia were implemented for confocal observation, biomass dry weight measurement, and RNA-seq analysis.

### Construction of *cel1b* overexpression and deletion strains

For *cel1b* overexpression in RUT-C30 using *tcu1* or *bxl1* promoters, the upstream sequences (~ 1500 bp) of *tcu1* or *bxl1* were amplified from the genomic DNA of *T. reesei* RUT-C30 and ligated to the plasmid p-DsRed [22] at *Spe*I and *Xba*I sites by the ClonExpress II One Step Cloning Kit (Vazyme Biotech Co., Ltd, Nanjing, China). The resulting expression vectors were ligated to *cel1b* at *Xba*I site and then transformed into RUT-C30 by *Agrobacterium*-mediated fungal transformation (AMT) method using hygromycin B as a marker [68], yielding recombinant strains OEcel1B-P_*tcu1*_ and OEcel1B-P_*bxl1*_, respectively.

For *cel1b* deletion in KU70, the upstream and downstream sequences (~ 1500 bp) of *cel1b* were separately amplified by PCR using genomic DNA of *T. reesei* KU70 as a template, and cloned into plasmid pXBthg at *Xho*I and at *Bam*HI utilizing ClonExpress™ II One Step Cloning Kit (Vazyme, China). The resulting plasmid pXBthg-cel1b was transformed into *T. reesei* KU70 by the AMT method using hygromycin B as a marker, yielding the deletion strain △cel1b. All the recombinant strains were confirmed by PCR amplification and sequencing. The PCR amplification result for confirmation of all the mutant strains were presented in Additional file 1: Fig. S2. The primers used were listed in Additional file 6: Table S5. PCR followed by sequencing was used to confirm the absence of *cel1b* and the correct integration of the deletion cassette (Additional file 1: Fig. S2D). The absence of the gene *cel1b* was tested by primers designed to amplify the coding sequence of *cel1b*. The 5′ integration was tested with the forward primer targeting the genome region outside of the upstream homologous sequence and the reverse primer targeted hygromycin B encoding gene *hph*. For testing 3′ integration, the forward primer was located at the gene *hph* and the reverse primer was designed to targeting the genome region outside of the downstream homologous sequence. The PCR product was further sequenced at Sangon Biotech to ensure the accuracy.

### Analysis methods

The (hemi)cellulase activities assay was performed as shown in our previous research [26, 35, 66, 69]. For sporulation assay, the spores of *T. reesei* RUT-C30 and OEcel1B were cultured in TMM + 2% cellulose agar plates and counted by a hemocytometer under a confocal microscope SP8 with a 20 × oil immersion objective. The fluorescence intensity of the culture supernatants was determined at excitation/emission wavelengths of 540/635 nm on a fluorescence spectrophotometer (Hitachi Ltd., Japan). For NaCl and CR sensitivity assays, the spores of *T. reesei* RUT-C30 and OEcel1B were inoculated on PDA plates with various concentrations of NaCl and CR and the diameters of colonies on plates were measured at indicated time points.

### Transcriptional analysis by qRT-PCR

Fresh mycelia were harvested at the indicated time points in the text. The total RNA was extracted with Fungi RNA Kit (Omega, R6840) following the protocol. The first-strand cDNA was synthesized using PrimeScript™ RT reagent Kit (Takara, RR047A). qRT-PCR was performed in an ABI 7500 real-time PCR system using TB Green Premix Ex Taq II (Takara, RR820Q). Analysis of the expression level was done using *sar1* gene as a reference. The relative mRNA level of each test gene was calculated by the 2 ^−ΔCt^ method, where ΔCt = Ct (test) − Ct (sar1). All the primers used are described in Additional file 6: Table S5.

### The biomass measurement of *T. reesei* grown on cellulose

The biomass of *T. reesei* grown in TMM + 2% cellulose was indirectly determined by the amount of intracellular protein [46, 47]. In brief, the mycelia of *T. reesei* were suspended in 1 M NaOH and incubated for 2 h with frequent vortex. Then the protein concentration of the supernatant of the suspension was determined by the Modified BCA Protein Assay Kit (Sangon Biotech, Shanghai, China). The biomass dry weight was calculated assuming an average content of 0.32 g intracellular protein per g of dry cell mass.

### Glucose and cellobiose uptake assay

For glucose and cellobiose assay, *T. reesei* RUT-C30 and OEcel1B were pre-cultured in SDB for 48 h and then were washed with the culture medium without any carbon source twice. After that, they were transferred to TMM + 1% glucose or cellobiose culture medium at 28 ℃ with 200 rpm. The residual glucose in the supernatant at different time points was measured using Glucose Detection Kit (Shanghai Rongsheng Biotech, China). The residual cellobiose in the supernatant at different time points was analyzed by HPLC with an Aminex HPX-87 H column.

### Confocal imaging of *T. reesei*

Mycelia of *T. reesei* RUT-C30 and its mutant strains were checked under a confocal microscope SP8 (Leica, Germany) with a 100 × oil immersion objective. The red fluorescence protein DsRed was observed at 570–700 nm with the excitation length of 552 nm. The green-emitting cell membrane/wall dye GC-PEG-cholesterol-FITC synthesized in our lab [48] was imaged in the emission range of 500–550 nm using the excitation wavelength of 488 nm. ER-Tracker (KeyGEN BioTECH Co. Ltd., China) was deployed to stain ER, which were carried out as described previously [22].

### Phylogenetic analysis of transporters

A data set containing 108 DEGs encoding membrane transporters from *T. reesei* was utilized for phylogenetic analysis. The amino acid sequences of these transporters from *T. reesei* and other species (*Saccharomyces cerevisiae* and *Neurospora crassa*) were obtained from the genome databases (https://fungi.ensembl.org/Trichoderma_reesei_rut_c_30_gca_000513815/Info/Index) and (https://www.ncbi.nlm.nih.gov/) respectively. Sequences were aligned and a phylogenetic tree was determined by neighbor-joining method using MEGA 5. Further visualization and annotation of the phylogenetic tree were performed by EvolView [70].

## Supplementary Information

**Additional file 1:Figure S1.** The mRNA level of *cel1b* in *T. reesei* RUT-C30 cultured in TMM + 2% cellulose for 120 h. Data are represented as the mean of three independent experiments, and error bars express the standard deviations. **Figure S2.** (A) Plasmid construction for *cel1b* overexpression. Kan: kanamycin resistance; LB, left border; RB, right border; Ptcu1 or Pbxl1, *tcu1* or *bxl1* promoter; Ttrpc, *Aspergillus nidulans* trpC terminator; linker, a short sequence linked *cel1b* and DsRed; hyg, hygromycin B phosphotransferase gene. (B) PCR confirmation of *cel1b* overexpression in strains OEcel1B-P_*tcu1*_ and OEcel1B-P_*bxl1*_. *T. reesei* RUT-C30 was used as a control. M: DL5000 DNA marker, 1: OEcel1B-P_*tcu1*_, 2: RUT-C30, 3: OEcel1B-P_*bxl1*_, 4: RUT-C30. (C) Schematic map of replacing gene *cel1b* with marker *hyg* by homologous integration in strain KU70, generating *cel1b* deletion strain △cel1b. (D) PCR confirmation of the knockout of *cel1b* in strain △cel1b. *T. reesei* KU70 was used as the control. The absence of the gene *cel1b* was tested by primers designed to amplify the coding sequence of *cel1b* (Primer 1b shown in Figure S2C). The 5′ integration was tested with the forward primer targeting the genome region outside of the upstream homologous sequence and the reverse primer targeted hygromycin B encoding gene *hph* (Primer up shown in Figure S2C). For testing 3′ integration, the forward primer was located at the gene *hph* and the reverse primer was designed to targeting the genome region outside of the downstream homologous sequence (Primer do shown in Figure S2C). **Figure S3.** (Hemi)cellulase activities and protein secretion for *T. reesei* RUT-C30 and *cel1b-*overexpressing strains using three different promoters grown in TMM + 2% cellulose for 168 h. Data are represented as the mean of three independent experiments, and error bars express the standard deviations. **Figure S4.** The mycelium length (A) and spore number (B) of *T. reesei* RUT-C30 and OEcel1B cultured in TMM + 2% cellulose for 168 h. Data are represented as the mean of three independent experiments, and error bars express the standard deviations. **Figure S5.** Cellulase activities and protein secretion for *T. reesei* RUT-C30 and OEcel1B grown in TMM + 1% cellobiose or TMM + 2% lactose. Data are represented as the mean of three independent experiments, and error bars express the standard deviations. **Figure S6** qRT-PCR analysis of genes involved in cellulase production, including cellulase genes (A), transcriptional factors (B), and sugar transporters (C). Strains KU70 and △cel1b were cultured in TMM + 2% cellulose for 72 h. **Figure S7.** The colony diameter (A), sporulation ability (B), and morphology (C) of *T. reesei* KU70 and *cel1b* deletion strain △cel1b. All strains were cultured on TMM liquid or agar plates with 2% cellulose. The spores were counted at 120 h and cell morphology observation was performed at 72 h by CLSM. Data are represented as the mean of three independent experiments, and error bars express the standard deviations. Scale bar = 10 μm. **Figure S8.** Fluorescence intensity of the supernatants from OEcel1B and RUT-C30 grown in TMM + 2% cellulose for 168 h. Data are represented as the mean of three independent experiments, and error bars express the standard deviations. **Figure S9** Confocal images of CEL1B-DsRed in strain OEcel1B cultured in TMM + 2% lactose. Scale bar = 10 μm. **Figure S10.** Confocal images of CEL1B-DsRed in strain OEcel1B cultured in TMM + 1% cellobiose. Scale bar = 10 μm. **Figure S11.** Sensitivity assay of the strains OEcel1B and RUT-C30 cultured in PDA agar plates with various concentration of Congo red (A) and NaCl (B). Data are represented as the mean of three independent experiments, and error bars express the standard deviations. **Figure S12.** Kyoto Encyclopedia of Genes and Genomes (KEGG) enrichment analysis of DEGs. The y axis represents the name of the most enriched pathways. GeneRatio: the number of DEGs in a specified GO term/ the number of the total DEGs in all GO terms; Count: the number of DEGs in a specified GO term; padj: *p* adjusted value.

**Additional file 2: Table S1.** Total DEGs in *T. reesei* OEcel1B as compared to RUT-C30.

**Additional file 3: Table S2.** DEGs involved in carbohydrate metabolic process.

**Additional file 4: Table S3.** DEGs involved in transmembrane transport process.

**Additional file 5: Table S4.** DEGs related to protein translocation across ER.

**Additional file 6: Table S5.** Primers for gene cloning, PCR confirmation and qPCR.

## Data Availability

The datasets supporting the conclusions of this article are included in the article and its Additional files.

## Abbreviations

CCR: Carbon catabolite repression
ER: Endoplasmic reticulum
pNPGase: The β-glucosidase activity
pNPCase: The CBH activity
CMCase: The CMC activity
FPase: The filter paper activity
pNPXase: The β-xylosidase activity
TMM: *Trichoderma* minimal medium
AMT: *Agrobacterium*-mediated fungal transformation
SDB: Sabouraud dextrose broth
PDA: Potato dextrose agar
HPLC: High-performance liquid chromatography
