# Supplementary material for: Transmembrane transport process and endoplasmic reticulum function facilitate the role of gene cel1b in cellulase production of Trichoderma reesei
Source: Microb Cell Fact. 2022 May 19;21:90. doi: 10.1186/s12934-022-01809-1 (PMC9118834; doi:10.1186/s12934-022-01809-1)
Supplement: Supplementary file 1 — Additional file 1:Figure S1. The mRNA level of cel1b in T. reesei RUT-C30 cultured in TMM + 2% cellulose for 120 h. Data are represented as the mean of three independent experiments, and error bars express the standard deviations. Figure S2. (A) Plasmid construction for cel1b overexpression. Kan: kanamycin resistance; LB, left border; RB, right border; Ptcu1 or Pbxl1, tcu1 or bxl1 promoter; Ttrpc, Aspergillus nidulans trpC terminator; linker, a short sequence linked cel1b and DsRed; hyg, hygromycin B phosphotransferase gene. (B) PCR confirmation of cel1b overexpression in strains OEcel1B-Ptcu1 and OEcel1B-Pbxl1. T. reesei RUT-C30 was used as a control. M: DL5000 DNA marker, 1: OEcel1B-Ptcu1, 2: RUT-C30, 3: OEcel1B-Pbxl1, 4: RUT-C30. (C) Schematic map of replacing gene cel1b with marker hyg by homologous integration in strain KU70, generating cel1b deletion strain △cel1b. (D) PCR confirmation of the knockout of cel1b in strain △cel1b. T. reesei KU70 was used as the control. The absence of the gene cel1b was tested by primers designed to amplify the coding sequence of cel1b (Primer 1b shown in Figure S2C). The 5′ integration was tested with the forward primer targeting the genome region outside of the upstream homologous sequence and the reverse primer targeted hygromycin B encoding gene hph (Primer up shown in Figure S2C). For testing 3′ integration, the forward primer was located at the gene hph and the reverse primer was designed to targeting the genome region outside of the downstream homologous sequence (Primer do shown in Figure S2C). Figure S3. (Hemi)cellulase activities and protein secretion for T. reesei RUT-C30 and cel1b-overexpressing strains using three different promoters grown in TMM + 2% cellulose for 168 h. Data are represented as the mean of three independent experiments, and error bars express the standard deviations. Figure S4. The mycelium length (A) and spore number (B) of T. reesei RUT-C30 and OEcel1B cultured in TMM + 2% cellulose for 168 h. Dat [file 12934_2022_1809_MOESM1_ESM.docx]

**Transmembrane transport process and endoplasmic reticulum function facilitate the role of gene *cel1b* in cellulase production of *Trichoderma reesei***

**Ai-Ping Pang,^a*^ Yongsheng Luo,^a*^ Xin Hu,^a^ Funing Zhang,^a^ Haiyan Wang,^a^ Yichen Gao,^a^ Samran Durrani,^a^ Chengcheng Li,^b^ Xiaotong Shi,^b^ Fu-Gen Wu,^a^ Bing-Zhi Li,^c^ Zuhong Lu,^a^**#  **Fengming Lin, ^a^**#

^a^State Key Laboratory of Bioelectronics, School of Biological Science and Medical Engineering, Southeast University, Nanjing, China

^b^International Innovation Center for Forest Chemicals and Materials and Jiangsu Co-Innovation Center for Efficient Processing and Utilization of Forest Resources, Nanjing Forestry University, Nanjing 210037, China

^c^Key Laboratory of Systems Bioengineering (Ministry of Education), School of Chemical Engineering and Technology, Tianjin University, Tianjin, China

#Address correspondence to Fengming Lin, linfengming@seu.edu.cn, or Zuhong Lu, zhlu@seu.edu.cn

**^*^**Ai-Ping Pang and Yongsheng Luo and contributed equally to this work and should be considered co-first authors.

**Additional file 1：**

**
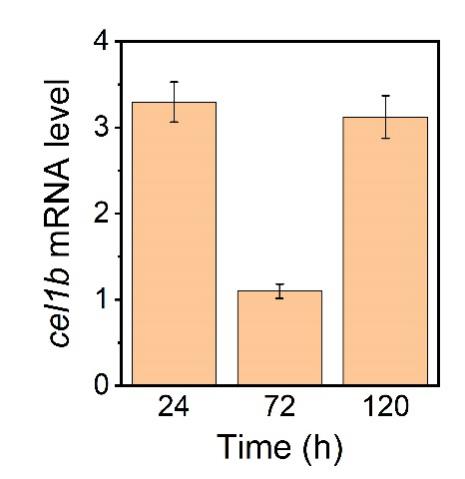
**

Figure S1 The mRNA level of *cel1b* in *T. reesei* RUT-C30 cultured in TMM + 2% cellulose for 120 h. Data are represented as the mean of three independent experiments, and error bars express the standard deviations.


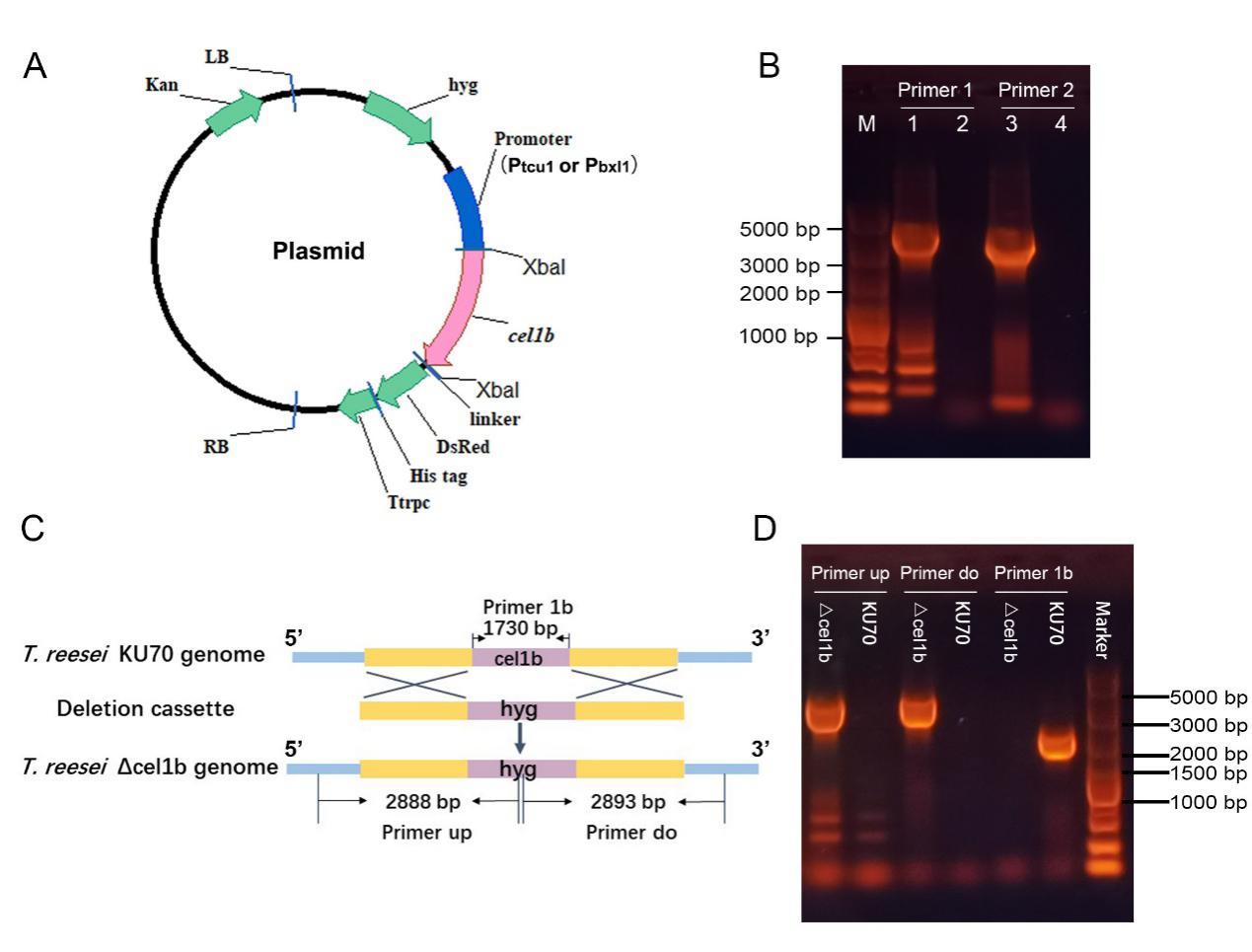


Figure S2 (A) Plasmid construction for *cel1b* overexpression. Kan: kanamycin resistance; LB, left border; RB, right border; Ptcu1 or Pbxl1, *tcu1* or *bxl1* promoter; Ttrpc, *Aspergillus nidulans* trpC terminator; linker, a short sequence linked *cel1b* and DsRed; hyg, hygromycin B phosphotransferase gene. (B) PCR confirmation of *cel1b* overexpression in strains OEcel1B-P*_tcu1_* and OEcel1B-P*_bxl1_*. *T. reesei* RUT-C30 was used as the control. M: DL5000 DNA marker, 1: OEcel1B-P*_tcu1_*, 2: RUT-C30, 3: OEcel1B-P*_bxl1_*, 4: RUT-C30. (C) Schematic map of replacing gene *cel1b* with marker *hyg* by homologous integration in strain KU70, generating *cel1b* deletion strain △cel1b. (D) PCR confirmation of the knockout of *cel1b* in strain △cel1b. *T. reesei* KU70 was used as the control. The absence of the gene *cel1b* was tested by primers designed to amplify the coding sequence of *cel1b* (Primer 1b shown in Figure S2C). The 5′ integration was tested with the forward primer targeting the genome region outside of the upstream homologous sequence and the reverse primer targeted hygromycin B encoding gene *hph* (Primer up shown in Figure S2C). For testing 3′ integration, the forward primer was located at the gene *hph* and the reverse primer was designed to targeting the genome region outside of the downstream homologous sequence (Primer do shown in Figure S2C).


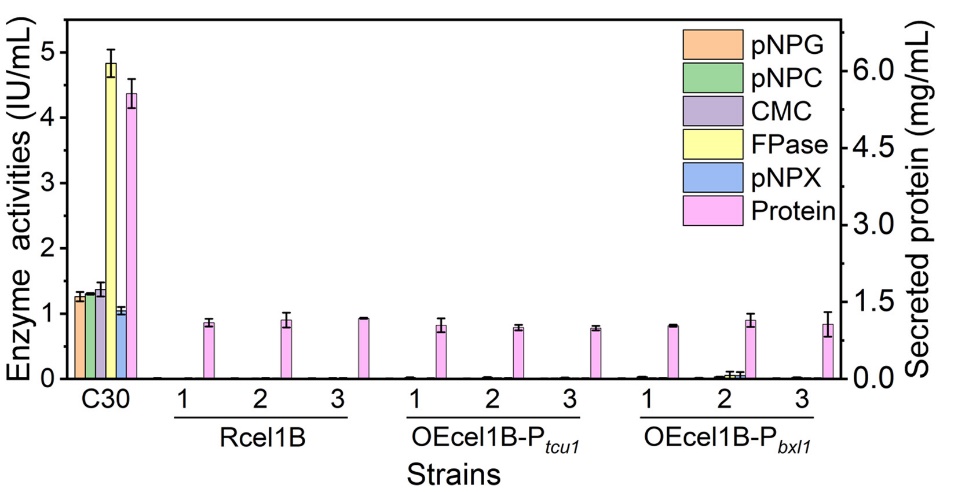


Figure S3 (Hemi)cellulase activities and protein secretion for *T. reesei* RUT-C30 and *cel1b-*overexpressing strains using three different promoters grown in TMM + 2% cellulose for 168 h. Data are represented as the mean of three independent experiments, and error bars express the standard deviations.


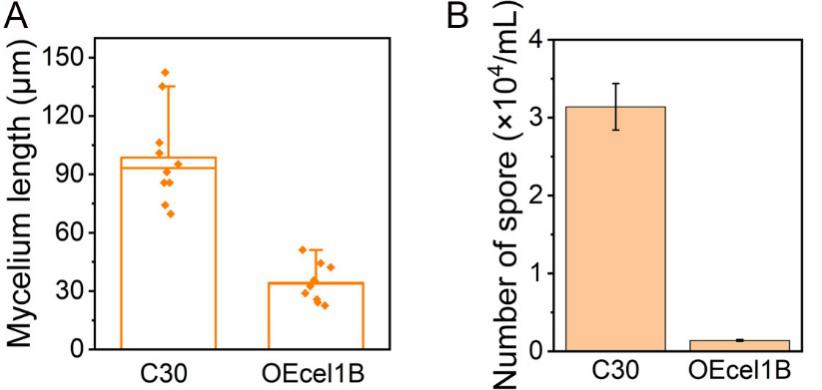


Figure S4 The mycelium length (A) and spore number (B) of *T. reesei* RUT-C30 and OEcel1B cultured in TMM + 2% cellulose for 168 h. Data are represented as the mean of three independent experiments, and error bars express the standard deviations.


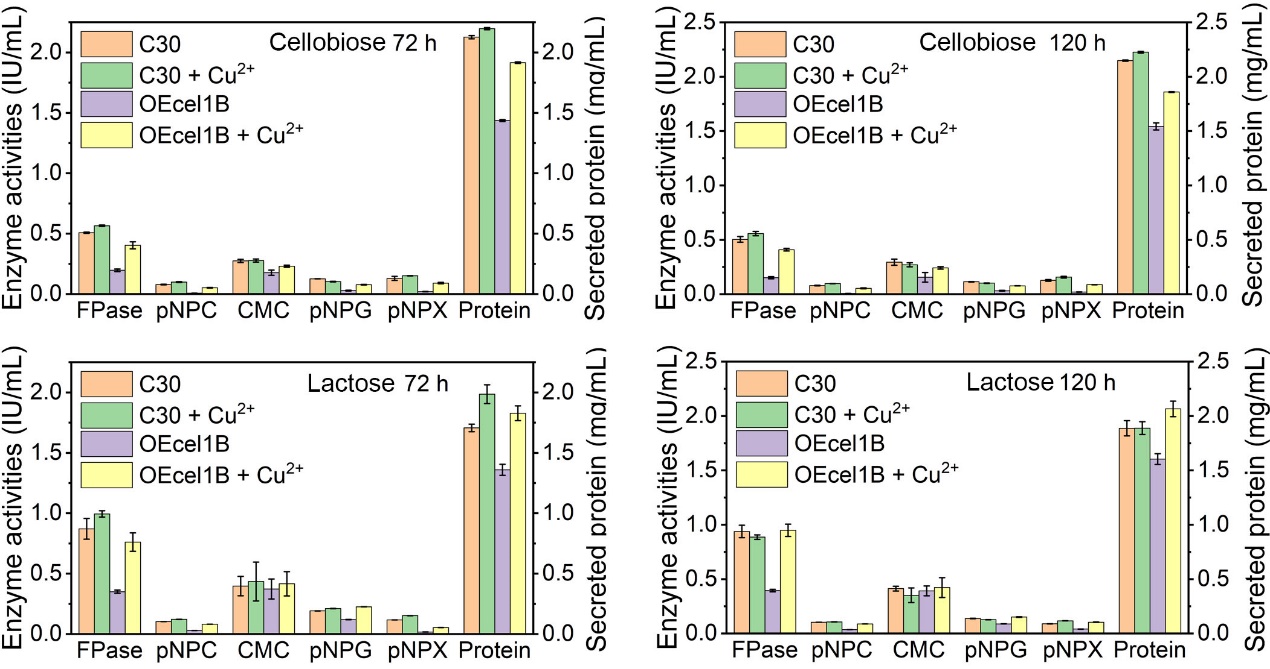


Figure S5 Cellulase activities and protein secretion for *T. reesei* RUT-C30 and OEcel1B grown in TMM + 1% cellobiose or TMM + 2% lactose. Data are represented as the mean of three independent experiments, and error bars express the standard deviations.


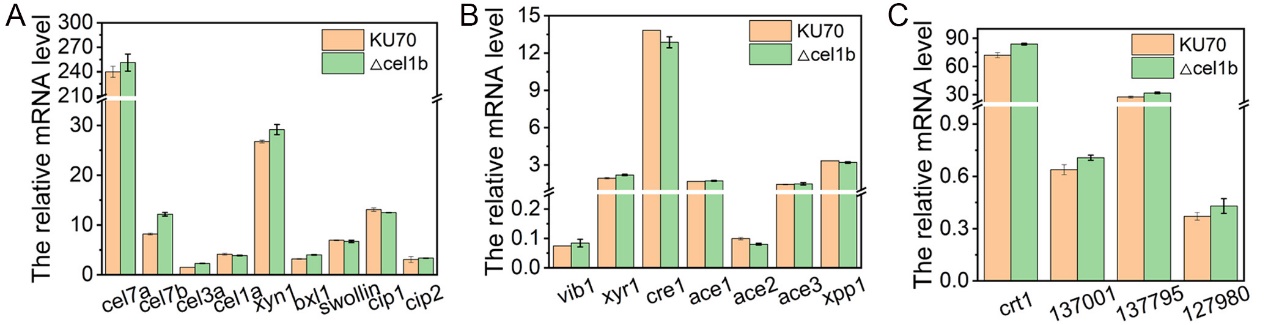


Figure S6 qRT-PCR analysis of genes involved in cellulase production, including cellulase genes (A), transcriptional factors (B), and sugar transporters (C). Strains KU70 and △cel1b were cultured in TMM + 2% cellulose for 72 h.


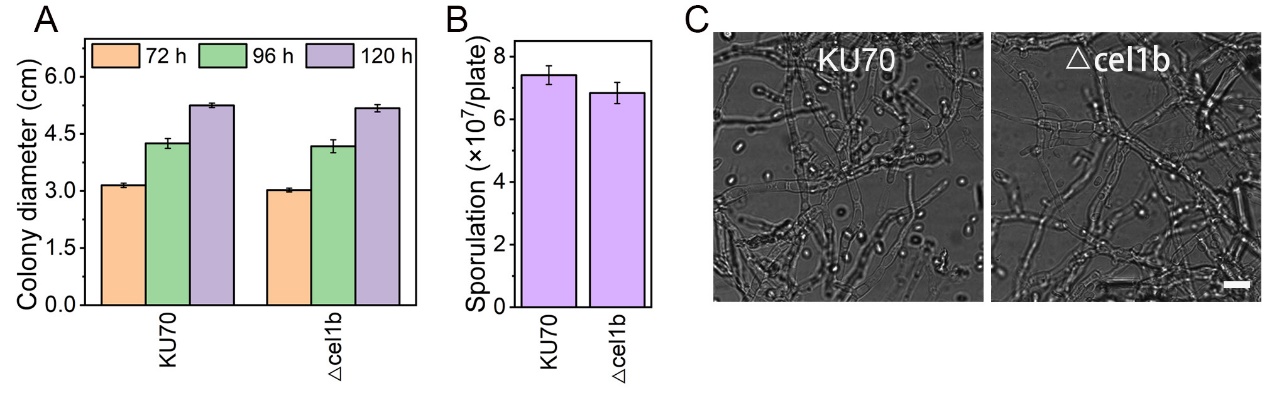


Figure S7 The colony diameter (A), sporulation ability (B), and morphology (C) of *T. reesei* KU70 and *cel1b* deletion strain △cel1b. All strains were cultured in TMM liquid or on agar plates with 2% cellulose. The spores were counted at 120 h and cell morphology observation was performed at 72 h by CLSM. Data are represented as the mean of three independent experiments, and error bars express the standard deviations. Scale bar = 10 μm.


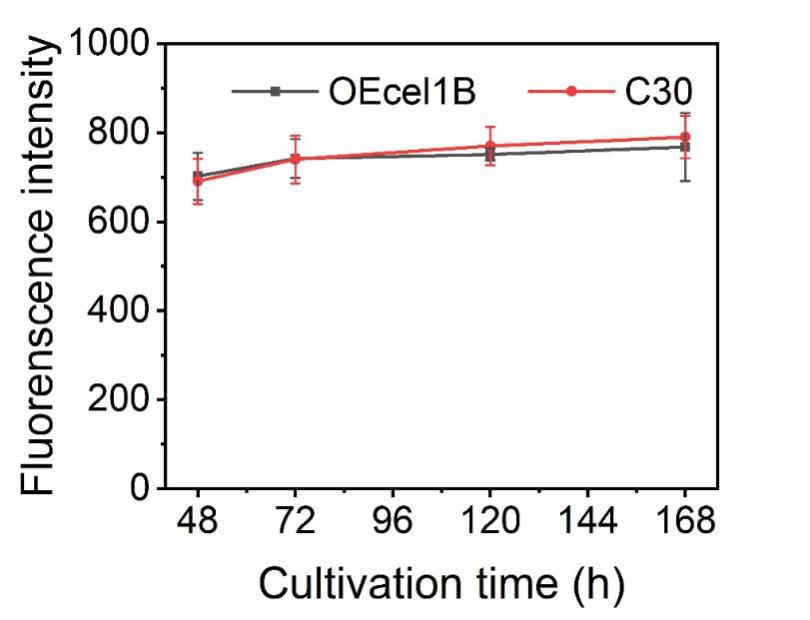


Figure S8 Fluorescence intensity of the supernatants from OEcel1B and RUT-C30 grown in TMM+2% cellulose for 168 h. Data are represented as the mean of three independent experiments, and error bars express the standard deviations.


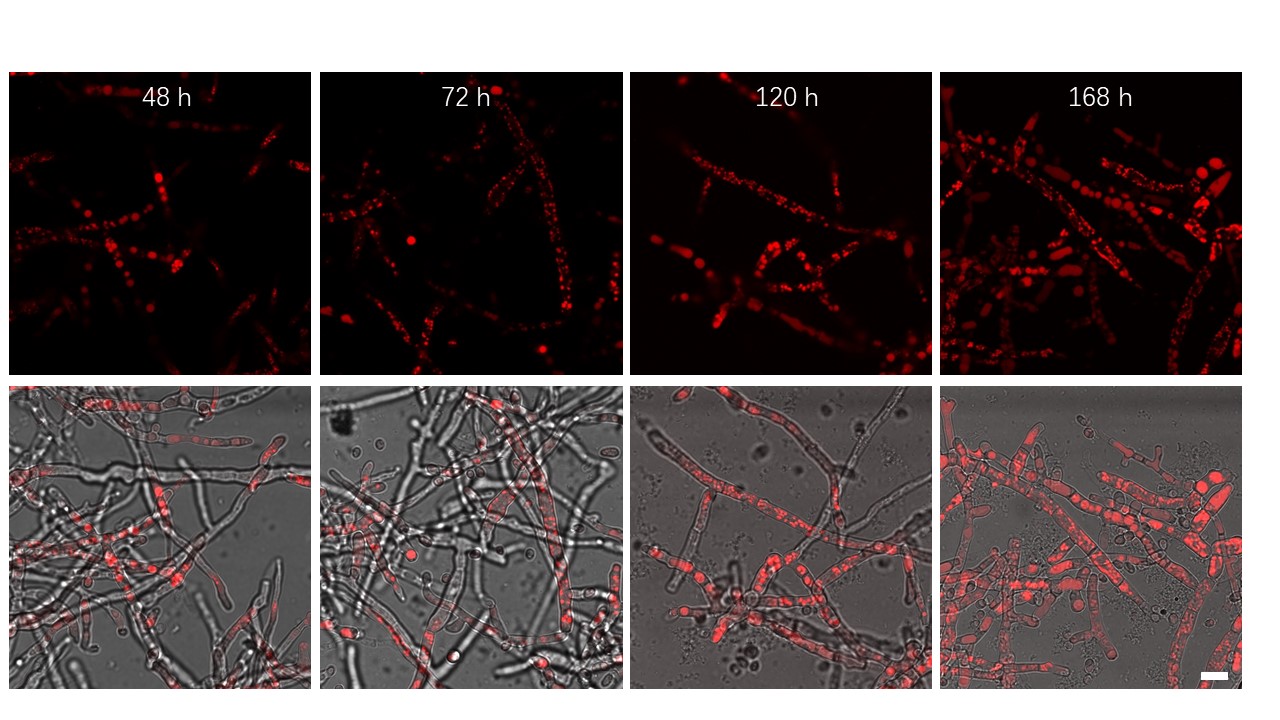


Figure S9 Confocal images of CEL1B-DsRed in strain OEcel1B cultured in TMM + 2% lactose. Scale bar = 10 μm.


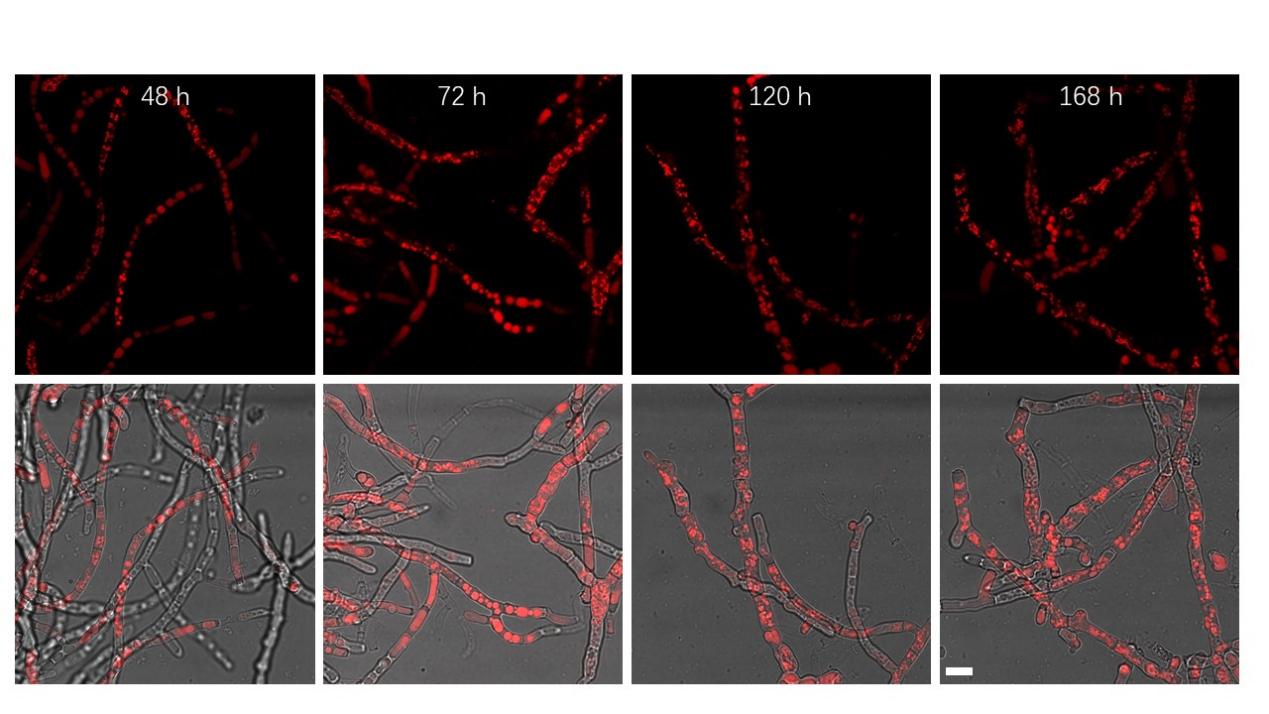


Figure S10 Confocal images of CEL1B-DsRed in strain OEcel1B cultured in TMM + 1% cellobiose. Scale bar = 10 μm.


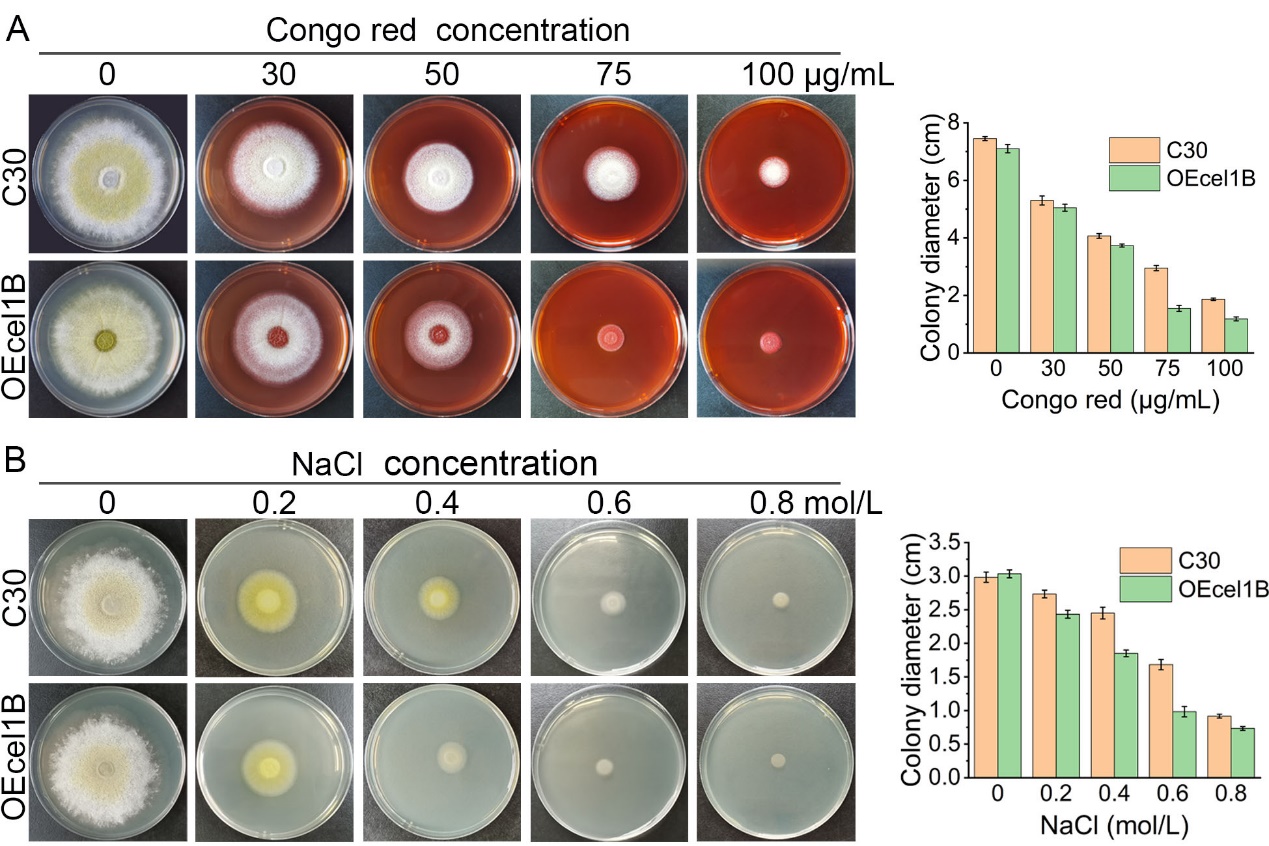


Figure S11 Sensitivity assay of the strains OEcel1B and RUT-C30 cultured in PDA agar plates with various concentration of Congo red (A) and NaCl (B). Data are represented as the mean of three independent experiments, and error bars express the standard deviations.


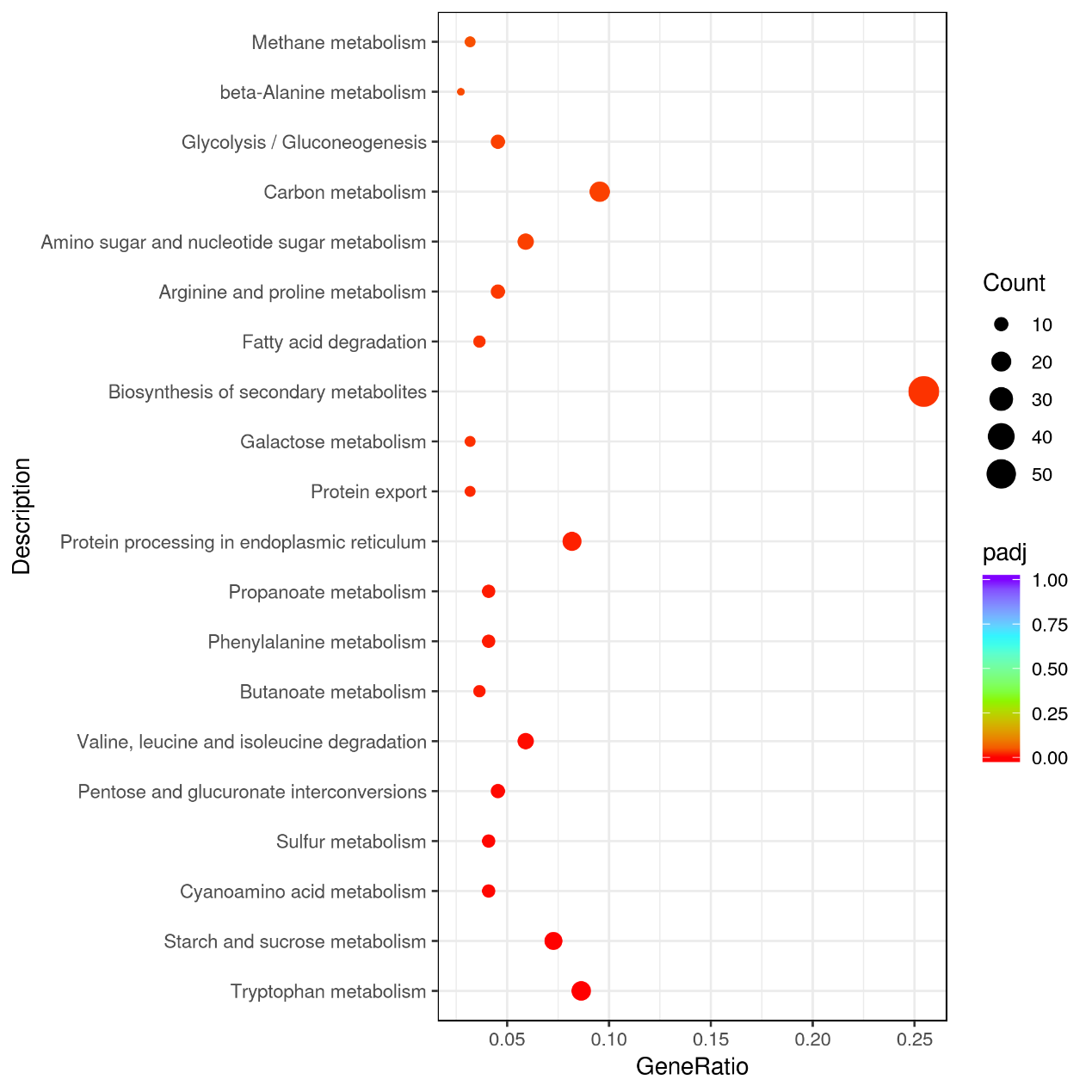


Figure S12 Kyoto Encyclopedia of Genes and Genomes (KEGG) enrichment analysis of DEGs. The y axis represents the name of the most enriched pathways. GeneRatio: the number of DEGs in a specified GO term/ the number of the total DEGs in all GO terms; Count: the number of DEGs in a specified GO term; padj: *p* adjusted value.
