# Supplementary material for: Transmembrane transport process and endoplasmic reticulum function facilitate the role of gene cel1b in cellulase production of Trichoderma reesei
Source: Microb Cell Fact. 2022 May 19;21:90. doi: 10.1186/s12934-022-01809-1 (PMC9118834; doi:10.1186/s12934-022-01809-1)
Supplement: Supplementary file 6 — Additional file 6: Table S5. Primers for gene cloning, PCR confirmation and qPCR. [file 12934_2022_1809_MOESM6_ESM.pdf]

Table S5 Primers used in this study

Primer	Sequence (5'– 3')
**For PCR cloning of *cel1b* overexpression and deletion in *T. reesei***	
tcu1-F	ACCAGCCCTGACTAGTGAGCGGAATCCTACATTCCCA
tcu1-R	CTGCTGCCGCTTCTAGAAGATCTTGTCGTATCAACCAGGTCGTATAG
bxl1-F	ACCAGCCCTGACTAGTTTCAGCCAGCGAATGAACGC
bxl1-R	CTGCTGCCGCTTCTAGATGCGTCCGGCTGTCCTTCA
tcu-1b-F	TGGTTGATACGACAAGATCTTCTAGAATGCCCGAGTCGCTAGCTCTG
tcu-1b-R	CTGCTGCTGCCGCTTCTAGATGCCGCCACTTTAACCCTCT
bxl1-1b-F	TGAAGGACAGCCGGACGCATCTAGAATGCCCGAGTCGCTAGCTCTG
bxl1-1b-R	CTGCTGCTGCCGCTTCTAGATGCCGCCACTTTAACCCTCT
cel1b-UP-F	ATTATTATGGAGAAACTCGAGCTGCCACGTCGGGAGAACTT
cel1b-UP-R	CCGTCACCAGCCCTGCTCGAGTTTTGAGCTATCTGGGTTGATTTCTC
cel1b-DO-F	GTGAGGGTTAATTGCGCGGATCCAAGAAAGGGAAATTTCTTCTTGCATTCAG
cel1b-DO-R	CAGGTCGACTCTAGAGAGGATCCCAGGCCAATGGCGAGCCAAA
**For PCR confirmation of *cel1b* overexpression and deletion in *T. reesei***	
Primer 1-F	ACCAGCCCTGACTAGTGAGCGGAATCCTACATTCCCA
Primer 1-R	TGCCGCCACTTTAACCCTCT
Primer 2-F	ACCAGCCCTGACTAGTTTCAGCCAGCGAATGAACGC
Primer 2-R	TGCCGCCACTTTAACCCTCT
Primer up-F	TCTCATCCGGCCCTTGGCAAT
Primer up-R	TCATTGACTGGAGCGAGGCGATGT
Primer do-F	GGGGATCAGCAATCGCGCATAT
Primer do-R	GGGTATCGAGGACGAGTTCCTGAG
Primer 1b-F	CCCGAGTCGCTAGCTCTG
Primer 1b-R	TGCCGCCACTTTAACCCTCT
**For qRT-PCR**	
Qsar1-F	TGGATCGTCAACTGGTTCTACGA
Qsar1-R	GCATGTGTAGCAACGTGGTCTTT
Qcel7a-F	GCGGATCCTCTTTCTCAGAC
Qcel7a-R	TTGGCGTAGTAATCATCCCA
Qcel7b-F	ACTACACGGAGGAGCTCGACGACTT
Qcel7b-R	AAGGCATTGCGAGTAGTAGTCGTTG
Qcel3a-F	TGACAGCTTCAGCGAGGGAC
Qcel3a-R	ACAAGACGGAGAGGCGTGAG
Qcel1a-F	CCTACCAGATCGAGGGCGC
Qcel1a-R	GCAGCGCAATGTCCTCGG
Qcel1b-F	TCGCACTTGGACTCGATTTCC
Qcel1b-R	CTTGAGGGTGGTGTAGTCTGTGAAC
Qxyn1-F	CTCCGTCAACTGGTCCAACT
Qxyn1-R	GTTGCCGTTTGGGTTGTAGC

Qbxl1-F	CGAGTTTGGCAGTGGTCTCT
Qbxl1-R	TGTGCGAACAAACAGCATGG
Qswollenin-F	CCGGAACCATGTATCCAGAGG
Qswollenin-R	GAGACCGTACAGGCCAAAGC
Qcip1-F	GGCAGCAACTCCATGAAGGT
Qcip1-R	CTGCCGAGAGCAGTCTGAAG
Qcip2-F	TGCTACGAGGACTTCGGCAT
Qcip2-R	CTTTGCGTTGGATCTCAGGG
Qcrt1-F	CCTTTTCCAGCTTTGCCACC
Qcrt1-R	CTCTTCCAAAGTGCGTCCCT
Q137795-F	AGGTCTCGCGGTGGTACTTG
Q137795-R	GCGTCGCCTCCTTGTTTCTC
Q127980-F	AGCATGCACTGCACTCCAGG
Q127980-R	ATGAGAGCACCTCGACAGGG
Q137001-F	GCTGGATCAGCGACAGATGC
Q137001-R	GAATCAGCGAGGACCCAACC
